# CAR-T cell potency: from structural elements to vector backbone components

**DOI:** 10.1186/s40364-022-00417-w

**Published:** 2022-09-19

**Authors:** Marzieh Mazinani, Fatemeh Rahbarizadeh

**Affiliations:** 1 Department of Medical Biotechnology, Faculty of Medical Sciences, Tarbiat Modares University, P.O. Box 14115-111, Tehran, Iran; 2 Research and Development Center of Biotechnology, Tarbiat Modares University, Tehran, Iran

**Keywords:** Chimeric antigen receptor, Cancer immunotherapy, Single-chain variable fragment, Nanobody, Lentiviral vectors, Signal peptide

## Abstract

Chimeric antigen receptor (CAR) T cell therapy, in which a patient’s own T lymphocytes are engineered to recognize and kill cancer cells, has achieved remarkable success in some hematological malignancies in preclinical and clinical trials, resulting in six FDA-approved CAR-T products currently available in the market. Once equipped with a CAR construct, T cells act as living drugs and recognize and eliminate the target tumor cells in an MHC-independent manner. In this review, we first described all structural modular of CAR in detail, focusing on more recent findings. We then pointed out behind-the-scene elements contributing to CAR expression and reviewed how CAR expression can be drastically affected by the elements embedded in the viral vector backbone.

## Introduction

**Fig. 1:**
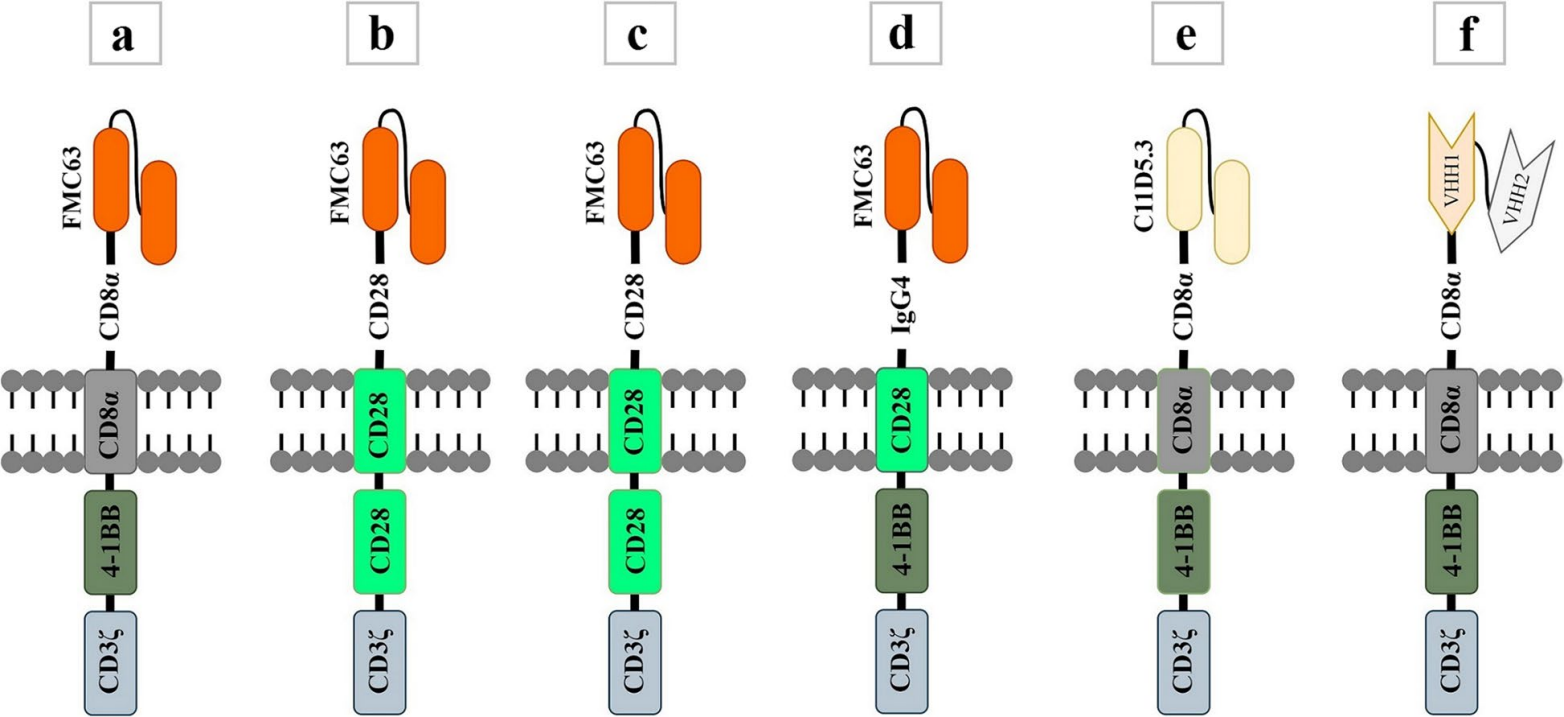
A schematic picture of the structural elements of six FDA-approved CAR-T products. Tisagenlecleucel (**a**), Axicabtagene ciloleucel (**b**), Brexucabtagene autoleucel (**c**), Lisocabtagene maraleucel (**d**), Idecabtagene vicleucel (**e**), Ciltacabtagene autoleucel (**f**)

Chimeric antigen receptor (CAR) T cell therapy, in which a patient’s own T lymphocytes are engineered to recognize and kill cancer cells, has achieved remarkable success in some hematological malignancies in preclinical and clinical trials, resulting in six FDA-approved CAR-T products currently available in the market [1–6] (Table 1) (Fig. 1). CARs are synthetic immune receptors that connect a single-chain variable fragment (scFv), derived from a monoclonal antibody, to T cell signaling domains to eradicate tumor cells independent of the major histocompatibility complex (MHC). Despite the impressive rate of complete remission (CR) in patients with certain B-cell malignancies [7–9], there are still some concerns about treatment failure associated with the low efficacy of CAR-T cells [10–13]. In the current review, we will discuss how the molecular components of CAR construct and elements of lentiviral vector backbone plasmid transferring CAR expression cassette can contribute to CAR-T cell therapy success or failure.

**Table 1:** FDA-approved CAR-T products

Generic Name	Tisagenlecleucel	Axicabtagene ciloleucel	Brexucabtagene autoleucel	Lisocabtagene maraleucel	Idecabtagene vicleucel	Ciltacabtagene autoleucel
Trade name	KYMRIAH**™**	YESCARTA**™**	TECARTUS**™**	BREYANZI®	ABECMA®	CARVYKTI
Company	Novartis	Gilead/Kite	Gilead/Kite	Bristol-Myers Squibb	Bristol-Myers Squibb	Janssen-Cilag International NV
Approval date	Aug-2017	Oct-2017	Jul-2020	Feb-2021	Mar-2021	Feb-2022
Target patients	R/R B-ALL R/R DLBCL	R/R DLBCL R/R NHL	R/R MCL	R/R LBCL	R/R MM	R/R MM
Targeted antigen	CD19	CD19	CD19	CD19	BCMA	BCMA

*R/R* Refractory/Relapsed, *ALL* Acute Lymphoblastic Leukemia, *DLBCL* Diffuse large B cell lymphoma, *NHL* Non-Hodgkin lymphoma, *MCL* Mantle cell lymphoma, *LBCL* Large B cell lymphoma, *MM* Multiple myeloma, *BCMA* B cell maturation antigen

## Structural elements contribute to CAR’s potency

Once equipped with a CAR, T cells, known as CAR-T cells, act as living drugs and recognize and eliminate the target tumor cells. The conventional CAR structure consists of three modular components: the ectodomain, the transmembrane domain, and the endodomain, each of which has specific components and functions and thus the potential to be optimized.

### Ectodomain

The ectodomain is the domain of a membrane protein that is outside the cytoplasm and exposed to the extracellular space. The ectodomain in the case of CAR consists of the antigen recognition region and hinge domain.

#### Antigen recognition domain

The predominant type of antigen-recognition domain in CARs is variable fragments of a conventional monoclonal antibody, commonly an IgG type, connected by a short peptide linker or disulfide bond and then called single-chain variable fragment or scFv [14]. Four crucial features of scFv that may affect CAR-T cell therapy clinical outcomes are affinity, immunogenicity, specificity, and structure. CAR binding affinity, along with its expression levels, determines the antigen-binding characteristics of the CAR and the efficacy of target cell recognition [15]. The CAR affinity must be sufficiently high to recognize the target antigen [16] but not too high to trigger on-target off-tumor toxicities [17]. It can be fine-tuned according to the target antigen density on tumor cells [18, 19]. Constructing CARs with the appropriate affinity to discriminate between malignant and normal cells without rendering any toxicity is crucial. Several studies have demonstrated that CAR with reduced affinity can efficiently distinguish tumors from normal tissues that express the same antigen at lower levels while maintaining potent antitumor activity and prolonged persistence [18–22].

Most CARs developed and tested in clinical studies have utilized scFvs, usually derived from murine monoclonal antibodies [23, 24]. Both humoral and cellular responses triggered by the murine-derived scFvs included in the CAR structure may lead to quick clearance of the CAR-T cells from circulation and thus increase the risk of relapse [25, 26]. Therefore, it seems that developing humanized or fully human scFvs, which are likely to be less immunogenic, would avert the anti-CAR responses and, consequently, circumvent treatment failure [27]. However, these scFvs may still have non-self-sequences since these variable fragments are usually generated through multiple recombination events and somatic hypermutation or fused at junctions that do not typically exist [25]. Lymphodepleting chemotherapy treatment before CAR-T cell infusion has a beneficial effect on reducing anti-CAR-T immune responses [28, 29].

**Fig. 2:**
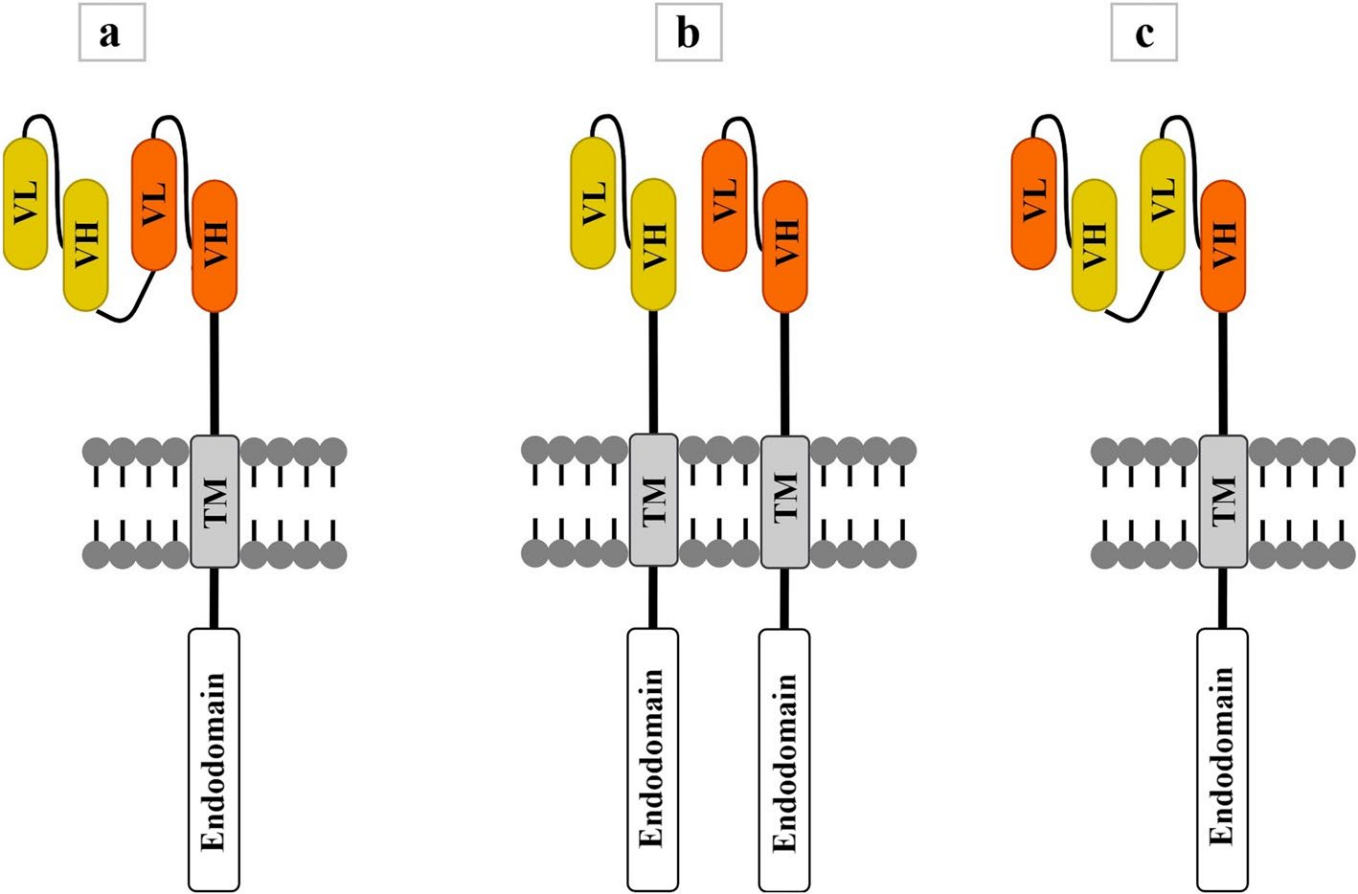
A schematic picture of TanCAR (**a**), DualCAR (**b**), and Loop CAR (**c**). In TanCAR, two different scFvs are connected outside the cell (in series), usually by a glycine-serine linker. TanCAR can be activated when any one of the scFvs binds to a target antigen. Dual CAR-T cells refer to the expression of two CARs in the same T cell, with each CAR having its own signaling function and distinct extracellular antigen recognition domains. Like TanCAR-T cells, loop CAR-T cells consist of two scFvs in a single CAR molecule. In TanCARs, the VL-VH of one scFv is directly linked to the VL-VH of the other scFv, whereas the loop structure is formed with the VL-VH of one scFv separated by the VL-VH of the other scFv

An ideal target antigen must be expressed with high specificity and coverage in tumor cells. Most antigens recognized by CARs are not tumor-specific (TSA), restricted to the tumor cells, but are tumor-associated (TAA), expressed on the surface of normal tissues as well, albeit at a low level. Targeting a TAA, in most cases, leads to unwanted on-target off-tumor toxicities, such as B cell aplasia, resulting from a direct attack on healthy tissues having a shared expression of the targeted antigen [30–32]. Therefore, finding a target with high specificity to tumor cells requires researchers to implement a comprehensive assessment. Tumor antigen heterogeneity, observed predominantly in solid tumors such as malignant mesothelioma (MM), glioblastoma multiforme (GBM), and so forth, is also one of the main impediments restricting the efficacy of monovalent immunotherapeutic strategies directed against only one particular antigen [33]. Therefore, efforts to develop CAR-T cell immunotherapy must confront this high diversity of potential target antigen expression; otherwise, treatment failure or tumor recurrence may occur [33]. Designing CARs with two scFvs in which two corresponding scFvs target two different antigens, such as tandem CARs (TanCAR), dual CARs, loop CARs, AND-gate CARs (synNotch-CAR), and inhibitory CARs (iCARs), is a common strategy to improve the specificity of CARs [30, 34]. In the TanCAR concept, also referred to as OR-gate CAR, two different scFvs are connected outside the cell (in series), usually by a glycine-serine linker [35, 36]. The TanCAR can be activated when any one of the scFvs binds to a target antigen. When two scFvs simultaneously bind to their respective target antigens, the TanCAR will be activated and produce synergistic effects, which results in further activation of CAR-T cells and boosting their tumor-killing ability [35–37]. Dual CAR-T cells refer to the expression of two CARs in the same T cell, with each CAR having its own signaling function and distinct extracellular antigen recognition domains [38–40]. Wang et al. showed that dual CAR-T cells targeting IL-23 and PSMA secreted more cytokines in vitro and functioned significantly better in mouse models of prostate cancer compared to TanCAR-T cells expressing the same scFvs in a single CAR [37]. Like TanCAR-T cells, loop CAR-T cells consist of two scFvs in a single CAR molecule. In TanCARs, the VL-VH of one scFv is directly linked to the VL-VH of the other scFv, whereas the loop structure is formed with the VL-VH of one scFv separated by the VL-VH of the other scFv [41]. It has been shown that loop CAR-T cells are more effective than the TanCAR-T cells in eradicating tumor cells and prolonging survival in xenograft models [42]. Loop CAR-T cells targeting CD19 and CD22 showed promising results in phase II clinical trial (NCT03196830) of patients with relapsed/refractory (R/R) non-Hodgkin lymphoma (NHL) [43] and in phase I clinical trial (NCT03233854) of adults with R/R acute lymphoblastic leukemia (ALL) and large B-cell lymphoma (LBCL) [44]. A schematic picture of TanCAR, dual CAR, and loop CAR has been illustrated in Fig. 2.

**Fig. 3:**
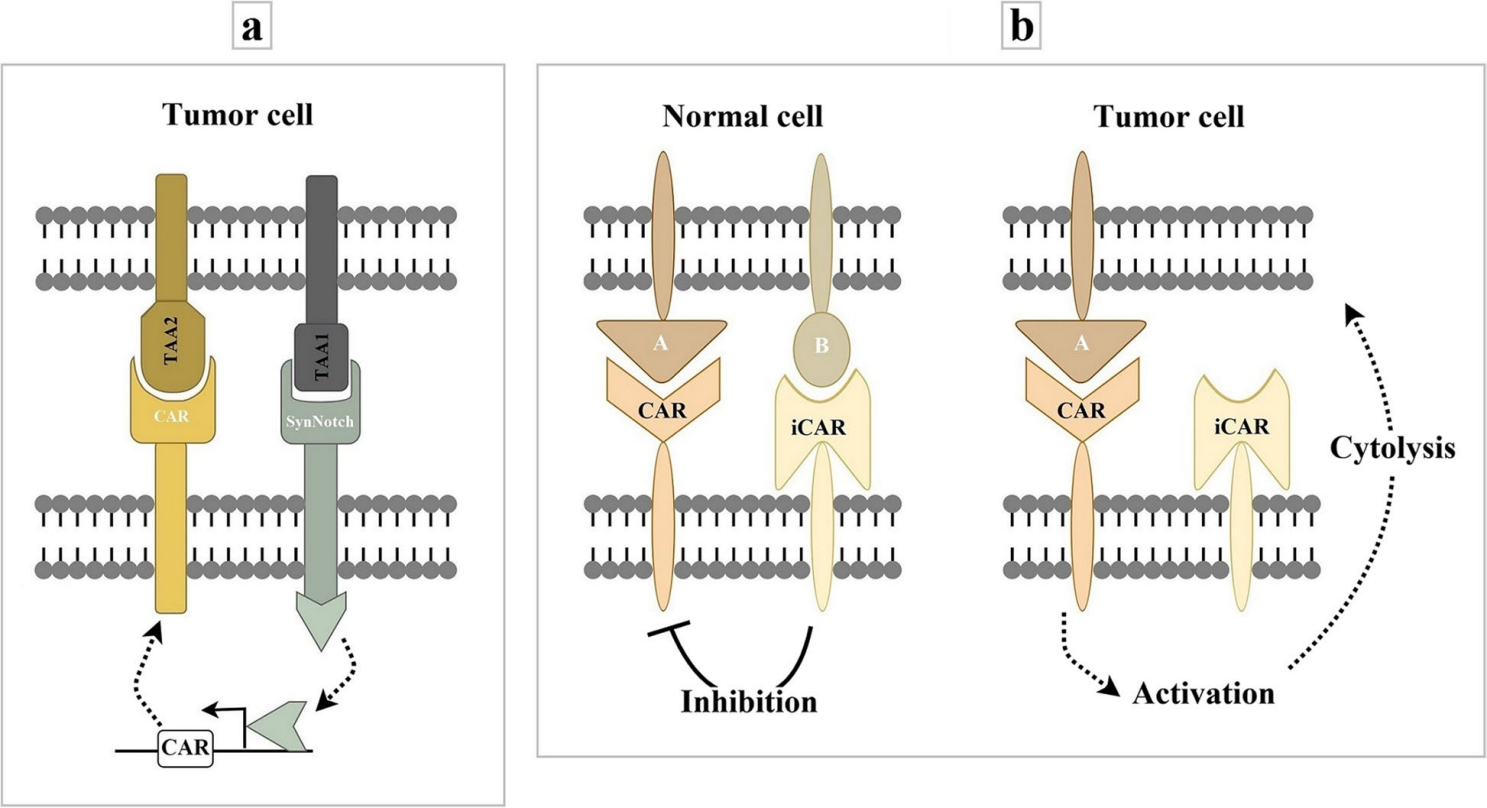
A schematic picture of synNotch-CAR (**a**) and iCAR (**b**). SynNotch-CARs require T cells to sense two antigens to activate. SynNotch receptors are engineered to sense a target antigen on the surface of tumor cells and induce the expression of a CAR specific to a second tumor antigen. iCAR-T cells express two CARs on the same T cells, including a typical tumor-antigen-specific CAR and an iCAR. Recognizing a target antigen on the surface of a normal cell by iCAR leads to the inhibition of the second CAR. iCAR, Inhibitory CAR; TAA, Tumor-Associated Antigen; A and B, Target Antigens

In contrast to OR-gate CARs, AND-gate CARs, with synthetic Notch (synNotch) receptors [45] as the core element, requires T cells to sense two antigens to activate (Fig. 3a). When the synNotch receptor recognizes a target antigen by its extracellular recognition domain, the transcriptional activator domain of the receptor is released, which can then enter the nucleus and drive the expression of a CAR for a second antigen. These combinatorially gated T cells has shown a remarkable degree of therapeutic discrimination both in vitro and in vivo [46]. Like dual CAR-T cells, iCAR-T cells express two CARs on the same T cells, including a typical tumor-antigen-specific CAR and an iCAR [47]. The iCAR consists of an scFv specific to the antigens expressed exclusively on normal tissue and an inhibitory signaling domain of immunoinhibitory receptors (programmed cell death protein-1 (PD-1) and cytotoxic T-lymphocyte-associated antigen 4 (CTLA-4)) to restrict T cell activity despite concurrent engagement of activating receptors, allowing T cells to distinguish target cells from the off-target cells [47, 48] (Fig. 3b).

ScFvs have a high propensity for self-aggregation, resulting from their structure, which leads to ligand-independent constitutive signaling, known as tonic signaling [49]. This tonic activation can induce early exhaustion of CAR-T cells and, consequently, limit its anti-tumor efficacy [50]. Amino acid point mutations or substitutions can partially correct the tonic signaling of CARs caused by its scFv [50, 51]. Although scFvs are currently the most often used antigen-recognition domain in CARs, some associated drawbacks, such as immunogenicity or their tendency for aggregation, may pose potential risks and challenges in their applications. Therefore, alternative antigen-binding domains would be beneficial [52]. The nanobody, also known as the VHH domain, is the variable domain of the heavy-chain-only antibodies (HcAb) naturally found in sera of camelids [53]. The VHH domain is the smallest fragment with antigen-binding capability, comparable to conventional antibodies in affinity and specificity [54]. Properties such as small size, high solubility and stability, low immunogenicity, high tissue penetration, and no need for the additional folding and assembly steps or linker optimization due to the lack of variable light chain make nanobodies a promising alternative to scFvs in CARs [54]. Using the VHHs as antigen recognition domains for CAR-T cells appears to be more favorable than scFv, particularly for solid tumors, as they can access epitopes hard or impossible to reach by scFvs [55–57]. The first report about the successful use of nanobodies in the CAR constructs emerged from our lab, where CAR-modified T cells used an anti-MUC1 VHH as the target-binding domain [55]. Anti-MUC1 CAR-T cells showed increased proliferation and IL2 secretion upon the stimulation and could effectively kill MUC1-positive tumor cell lines [55]. For creating more complicated CARs, nanobodies are more favorable than scFvs, due to their compact size and lack of VL chain. Furthermore, the potential cross-pairing of VH and VL, commonly observed among two independent scFv molecules but not among nanobodies, may result in the affinity loss of these scFv-based CARs [58]. Interestingly, the sixth CAR-T product, recently approved by the US FDA for medical use in patients with R/R multiple myeloma (MM), utilizes two VHHs targeting two different B-cell maturation antigen (BCMA) epitopes [59] (Fig. 1). Search for finding antigen binding domains other than scFv has led to new ligand-based CARs. Recently, Wang et al. used the intrinsic binding properties of a natural toxin to develop targeted CAR-T cells. They incorporated chlorotoxin (CLTX), a 36-amino acid peptide isolated from the deathstalker scorpion venom previously used to deliver radiation therapy and imaging reagents to tumor sites of patients with GBM, into a CAR construct to redirect cytotoxic T cells against GBM cells. They found the antitumor effects of CLTX-CAR-T cells robust and specific while making negligible off-tumor toxicity [33]. Zetakine-CARs employ a membrane-tethered cytokine ligand as the antigen recognition domain [60, 61]. Brown et al. developed a zetakine-CAR targeting IL-13 receptor α2 (IL-13Rα2) by fusing a membrane-tethered IL13 ligand mutated at a single site (E13Y) domain to intracellular signaling domains to use for the treatment of recurrent GBM [62]. Moeller et al. also developed an IL3- specific zetakine-CAR targeting the alpha-chain of the IL3 cytokine receptor (CD123), a promising target for acute myeloid leukemia (AML) as it is overexpressed in both leukemic stem cells (LSCs) and blasts [61]. Other ligand-based CARs have been developed and tested in preclinical and clinical studies across a range of malignancies, including those incorporating FMS-like tyrosine kinase 3 ligand (FLT3L) to target FLT3-positive AML [63, 64], Natural Killer Group 2D (NKG2D) receptor to target NKG2D ligands on the surface of tumor cells [65], a proliferation-inducing ligand (APRIL), also known as TNFSF13, to target BCMA and transmembrane activator and calcium-modulator and cyclophilin ligand (TACI), two receptors implicated in the pathogenesis of multiple myeloma [66], and granulocyte-macrophage colony-stimulating factor (GM-CSF) to target the GM-CSF receptor (CD116) involved in the pathogenesis of juvenile myelomonocytic leukemia (JMML) [67].

#### Linker

The antigen-binding domain of CARs is composed of two distinct critical components; the scFv and the linker, whose rational design is often neglected while designing a CAR construct. The content and length of linkers strongly influence the structural and functional properties of CARs. Although linkers are highly divergent in their sequences, those commonly used in CARs contain repeats of glycine (Gly) and serine (Ser) residues to provide the flexibility necessary for antigen-binding sites to change conformation [68] and maintain good stability in aqueous solutions [69]. The combination of Gly and Ser residues also prevents the formation of secondary structures and reduces the likelihood of the linker interfering with the folding and function of the scFv [70]. The length of the linker between V-domains affects various structural characteristics of scFv, such as size, flexibility, and valency. A linker greater than 12 residues helps V-domains fold in the natural orientation and form a monovalent antigen-binding site [71]. In contrast, shorter linkers restrict V-domain flexibility, prevent intramolecular variable chain pairing, and instead favor intermolecular oligomerization and formation of scFv multimers [72–74]. A recent study, through detailed interrogation, demonstrated how linker length drastically affects the clinical outcomes of CAR-T cell therapy. Singh et al. set out a study at the University of Pennsylvania (Penn)/ Children’s Hospital of Philadelphia (CHOP) to evaluate CD22 as a target for CAR-T cell therapy. After analyzing the results of two pilot clinical trials (NCT02650414 and NCT02588456) using anti-CD22 CAR-T in six pediatric and three adult patients with R/R ALL, they found that response rates were unexpectedly lower compared with results from a similar trial at the National Cancer Institute (NCI) [11, 75]. Further investigation revealed similar patient characteristics, manufacturing processes, and CAR constructs in these clinical trials; the only difference was the length of the scFv linker; the anti-CD22 CAR evaluated by the NCI joined the V-domains using a five-amino acid linker ((Gly4Ser), CAR22-short), while the trials at the Penn / CHOP connected the V-chains using a 20-aa linker ((Gly4Ser)_4_, CAR22-long) [76]. They found that the CAR22-long was monomeric, distributed uniformly on the cell surface, while the CAR22-short was homodimerized and clustered on the cell surface. The CAR22-short demonstrated greater F-actin polarization and perforin accumulation at the immune synapse (IS), more durable immune synapse formation, and enhanced downstream receptor activation. The CAR22-short also secreted more IFN-γ and drove enhanced cytotoxicity against target tumor cells than its long counterpart. Antigen-independent signaling associated with linker-driven clustering causes CAR22-short T cells to be ‘primed’ and rapidly initiate intracellular signaling and activate immune response programs upon target engagement. Interestingly, the tonic signaling associated with the shortened linker was beneficial for CD22 CARs bearing the 4-1BB costimulatory domain, while replacing 4-1BB with CD28 led to T cell exhaustion and dysfunction. Furthermore, they found that the association between linker length and clustering is not a universal phenomenon. In CARs targeting CD19, shortening the scFv linker did not result in cell surface clustering and tonic signaling [76].

#### Hinge domain

A domain usually overlooked while evaluating CAR functionality is the hinge domain (HD). The HD in CARs serves as a spacer that holds scFvs beyond the plasma membrane and gives them the flexibility necessary to access antigen epitopes on the surface of target cells [77, 78]. The origin, length, flexibility, and composition of the HD influence CAR antitumor activity and the occurrence of side effects as well [79–81]. The HDs typically used in CARs are Ig-based, originating from the constant region of human immunoglobulin molecules, or non-Ig based derived from the components naturally expressed on T cells. Ig-based spacers commonly use the hinge-CH2-CH3 regions of IgG molecules, mostly IgG1 and IgG4 [82–85]. Accumulated research studies have shown that effective antigen recognition by CAR relies on the length of HD and the target epitope distance from the cell membrane [78, 86, 87]. A study by Qin et al. showed that hinge-containing anti-CD19 CAR-T cells had a tumor-eradication capacity similar to their hinge-free counterparts. These results suggested that for those antigen epitopes like CD19, which are membrane-distal and expressed on the cell surface with high density, embedding a hinge domain in CAR construct does not improve their killing activities [78]. However, they found that hinge incorporation improved the expansion and migratory capacity of anti-CD19 CAR-T cells. They also achieved similar results with anti-mesothelin CAR-T cells [78]. To access antigen epitopes residing closer to the membrane of target cells or those embedded within heavily glycosylated structures, a hinge domain with proper length is needed to decrease the distance or amend the steric inhibitory effects between the scFv and its epitope [78, 86]. Hudecek et al. designed three CAR constructs bearing either a full-length or truncated IgG4-Fc as a spacer to study the effect of HD length on CAR antitumor activity [87]. All CAR constructs recognize a membrane-distal epitope of the receptor-tyrosine kinase-like orphan receptor 1 (ROR1). They found that HD length did not affect the CAR expression levels; however, CARs with intermediate and short spacer showed superior T-cell cytokine secretion and proliferation after target-antigen recognition [87]. Therefore, the HD length in CARs needs to be carefully tailored regarding the target epitope distance from the cell membrane to achieve an improved antitumor efficacy.

Several clinical studies have reported a lack of persistence in CAR-T cells bearing IgG-derived spacers [82, 88]. Studies have shown that the IgG-derived spacers have ligand-binding capacity. Several amino acid sequences within the CH2 domain can bind to Fcγ receptors (FcγRs) on the innate immune cells, including monocytes/ macrophages, dendritic cells (DC), neutrophils, and natural killer (NK) cells [89]. The Fc: FcγR binding may result in unwanted innate immune response, including antibody-dependent cell-mediated cytotoxicity (ADCC) and phagocytosis, which may, in turn, result in depletion of CAR-T cells having Ig-based spacers in their CAR constructs [90, 91]. The Fc: FcγR interaction may also lead to ligand-independent tonic signaling and subsequently activation-induced T-cell death (AICD) [90, 92]. Therefore, modification of the IgGs-derived spacers, such as replacing the IgG1-CH2 framework with the corresponding IgG2 amino acids [26, 29], which has a lower binding capacity to both human [32] and murine [31] FcγRs, or complete deletion of CH2 region may solve the problems [22]. Jonnalagadda et al. generated several anti-CD19 CARs with different IgG4-derived spacers; one with a nonmutated CH2 domain, one with complete deletion of the CH2 region, and the others with single- or double-point mutations in the CH2 region. They found that CAR with nonmutated CH2 failed to engraft and persist in xenograft models, probably due to Fc binding to FcγR. However, the engraftment and persistence were partially restored by blocking this interaction using intravenous immunoglobulin (IVIG) administration [91]. Moreover, they observed that double point mutations or complete deletion of the CH2 region resulted in an improved persistence and antitumor efficacy in xenograft models compared with CARs containing a nonmutated or single-mutated CH2 [91]. Thus, introducing mutations, truncations, or complete deletion in the IgG-derived spacers is inevitable to diminish the adverse consequences of Fc: FcγR binding and improve CAR persistence and efficacy in vivo [91, 93]. However, CAR-T cells with no in vivo therapeutic efficacy or persistence were reported even after similar modifications in their IgG1-derived spacers [94]. Breyanzi, the FDA-approved CAR product of Juno Therapeutics/Bristol Meyers Squibbs, of note, harbors a 12-amino acid IgG4-derived spacer without the CH2-CH3 sequence (IgG4 hinge only) [93, 95].

To minimize the possibility of potential immunological interactions elicited by the Ig-based spacers and attain the safety needed for clinical use, spacers derived from components naturally expressed on T cells, such as CD8 and CD28, can be incorporated into CAR structure [96]. Alabanza et al. investigated the effect of the hinge and transmembrane (TM) domains derived from human CD28 or CD8α on the biology of fully human or murine-derived CD28-based CARs targeting CD19. They found that regardless of scFv origin, both CARs with CD8α or CD28 HD/TMD showed similar expression levels on the T cell surface. Compared with CARs containing HD/TMD of CD8α, those with CD28 HD/TMD produced significantly higher inflammatory cytokines and underwent more AICD [79]. T cell exhaustion markers such as PD-1 and lymphocyte activation gene-3 (LAG-3) were also higher in CAR-T cells containing CD28 HD/TMD. Based on crystal structures, they realized that enhanced inflammatory cytokine production and AICD observed in CAR-T cells with CD28 HD/TMD resulted from an increased tendency of these CARs to dimerize compared with CARs containing CD8α HD/TMD. They also found that HD/TMD does not affect T cell memory phenotype [79].

In patients with a high tumor burden, CAR-T cell therapy may result in adverse reactions and side effects, such as cytokine release syndrome (CRS), associated with the over-activation of CAR-T cells [97–99]. Considering that the spacer appears to be involved in T cell activation and cytokine production, its modification may provide the safety and efficacy needed for patients with a high tumor burden. To decrease the over-activation of CAR-T cells, Zhang et al. removed two consecutive Gly residues in the CD8-derived spacer of a second-generation anti-CD19 4-1BB-based CAR to reduce spacer flexibility. They found that this modification resulted in better tumor control and lower release of inflammatory cytokines in vivo. Also, they observed a downward trend in tumor load and prolonged survival in xenograft models treated with CAR-T cells bearing a less flexible spacer [100]. In an endeavor to develop long spacer domains with a favorable functionality profile for membrane-proximal targets, Schafer et al. introduced a novel class of spacer derived from the Sialic acid-binding immunoglobulin-type lectins (Siglecs). A long spacer derived from Siglec showed potential cytotoxicity, and its performance was similar to the CD8α spacer in a CAR targeting CD20 in vitro and in vivo while maintaining a favorable cell phenotype profile and cytokine release pattern [94].

The spacer in the CAR construct can also be used for identification, purification, and in vivo tracking of CAR-positive subsets of T cells after engineering [96, 101]. Casucci et al. demonstrated that the incorporation of the nerve growth factor receptor (NGFR) as a spacer into the CAR backbone enables the enrichment of CAR-T cells before infusion into patients and facilitates the in vivo tracking, phenotypic characterization, and isolation of CAR-T cells for ex vivo analysis [101]. They also showed that NGFR, when incorporated into the CAR molecule, cannot trigger signaling upon in vivo growth­ factor encounter [101]. Bister et al. also inserted a CD34-derived spacer into the CAR backbone to facilitate the detection and enrichment of CAR-T cells before infusion. This spacer was functionally similar to the CD8 spacer in in-vitro and in-vivo experiments [96].

Consistent with studies concerning scFv-based CARs, spacers can affect the expression level and functional activity of nanobody-based CARs [102]. In our previous study, we incorporated three different spacers, including CH3–CH2-hinge and CH3–CH2-hinge-hinge regions derived from human IgG3 and the hinge region of the FcγRII, into anti-MUC1 CARs and observed a greater expression of CARs containing those spacers derived from human IgG3. We also observed that CARs having two repeats of hinge sequence in their spacers (CH3–CH2-hinge-hinge) showed more flexibility which may induce homodimerization and increase the avidity of CAR for the target antigen [102, 103].

### Transmembrane domain

The transmembrane domain (TMD), like the hinge domain (HD), is a component in the CAR structure that connects the antigen recognition moiety to the intracellular signaling domain. It is mainly derived from type-I single-spanning proteins, such as CD3ζ, CD4, CD8α, or CD28. The TMD is primarily considered a structural block in the CAR that anchors the receptor in the cell membrane. However, the functional importance of TMD in CAR expression level and stability has been well-established [104, 105]. Fujiwara et al. studied the effect of HD and TMD, derived from various molecules, such as CD4, CD8α, or CD28, on the expression level and antigen-specific cytotoxic activity of CAR [104]. They found that the CAR expression level was enhanced much more in HD/TMD-modified than in HD-modified CARs, suggesting that CAR expression level and stability on the T cells were highly affected by TMD rather than HD [104]. A high surface expression was also reported by Zhang et al. following the incorporation of either CD8α or CD28 TMD into the CAR construct [106]. The TMD of CARs mediates CAR dimerization and interaction with endogenous proteins, forming dimers or trimers [79, 80, 105]. Annenkov et al. showed that CARs containing the transmembrane region of FcεRIγ mediate T cell activation by heterodimerizing with CD3ζ [107]. Bridgeman et al. demonstrated that embedding the transmembrane region of CD3ζ in the CAR structure facilitates signal transmitting and T cell activation via mediating the homodimerization of chimeric receptors or their interaction with the endogenous TCR [105, 108]. Alabanza et al. also showed that CD28-derived HD/TMD has a greater tendency to drive homodimer formation compared to CD8α HD/TMD. This homodimerization can cause an increased tonic signal and AICD in T cells expressing CD28-HD/TMD CARs [79]. Muller et al. found that CD28-TMD can also interact with the endogenous CD28 receptor and form CD28-CAR heterodimers [80]. The CD28-CAR dimers may cause higher on-target off-tumor toxicities by enhancing CAR sensitivity to ectopically expressed low-density antigens, such as the CD19 on brain mural cells [80, 109]. Nevertheless, Majzner et al. demonstrated that the CD28 HD/TMD provides a more stable and efficient immune synapse and decreases the antigen-density threshold for T-cell activation in CD19-specific CARs compared to their CD8 counterparts [110].

The CAR hinge and transmembrane regions can also influence CAR-T cell cytokine production [81]. To find how HD/TMD affects the expansion, cytokine production, and memory generation of CAR-T cells, Ying et al. incorporated CD8α-derived HD/TMD with different lengths into CAR. They found that a CAR harboring an 86-amino-acid HD/TMD produces potent antitumor responses without enhancing serum cytokine concentrations responsible for CRS and neurological toxicity. Thus, their results suggest that modification of CAR hinge and transmembrane regions can modulate cytokine secretion and help ameliorate CAR-T cell-associated toxicities [81]. Guedan et al. also showed that a third-generation CAR composed of ICOS and 4-1BB intracellular domains (ICDs) displayed superior antitumor activity and increased persistence in vivo only when the ICOS ICD was directly fused to an ICOS transmembrane domain [111]. The TMD of ICOS has a constitutive, albeit weak, association with the tyrosine kinase Lck. This association facilitates p85 recruitment to ICOS and subsequent PI3K activation. When incorporated into the CAR, ICOS TMD augments the proximal signaling output by providing an extra pool of Lck [112].

The TMD in the CAR structure can be designed according to the transmembrane-mediated interaction and functionality desired. Schmith et al. used the TMD of 4-1BB to form trimeric CARs, thus enhancing the antigen-binding capacity of the CAR and reducing antigen escape [113]. Wang et al. also generated a novel chimeric receptor in which the transmembrane and cytoplasmic domains of KIR2DS2, a stimulatory killer immunoglobulin-like receptor (KIR), expressed naturally in CD4 and CD8 T and NK cells, fused to an scFv. They then introduced this KIR-based CAR into human T cells expressing DAP12, an immunoreceptor tyrosine-based activation motif (ITAM)-containing adaptor. They found that T cells expressing KIR-CAR fail to trigger cytotoxic activity without KIR/DAP12 association. They also found that the KIR-based CAR exhibited more potent antitumor activity in vivo compared with 2nd-generation CD3ζ-based CARs due to enhanced stability of the KIR/DAP12 complex within the plasma membrane following antigen engagement [114].

### Endodomain

#### Costimulatory domain

**Fig. 4:**
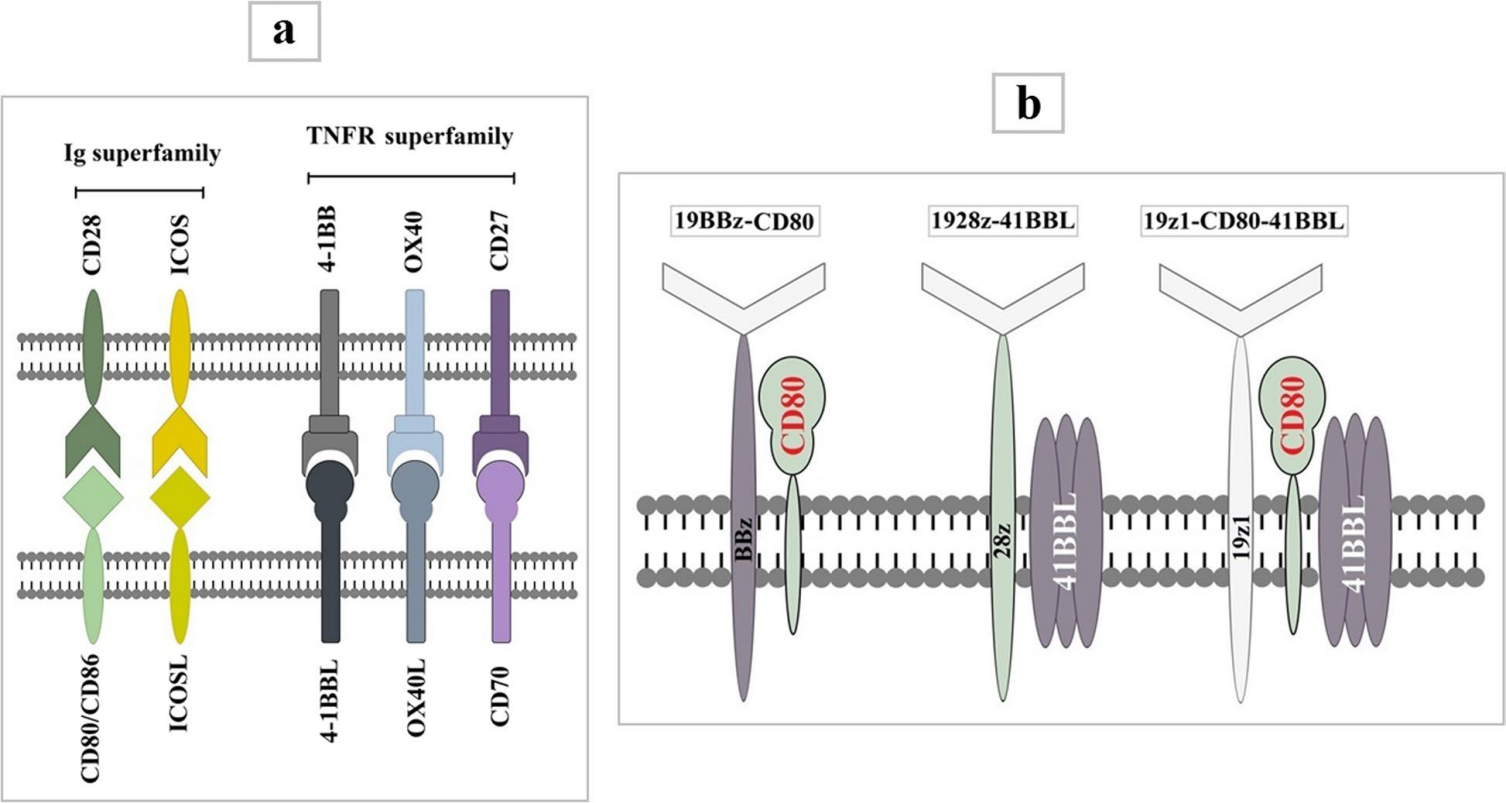
A schematic picture of costimulatory molecules commonly used in CAR construct and their ligands on antigen presenting cells (**a**), Three CAR configurations (19BBz-CD80, 1928z-41BBL, and 19z1-CD80-41BBL) in Zhao’s study that are coupled with complementary costimulatory ligands (**b**). The 19BBz-CD80 and 1928z-41BBL are two 2nd-generation CARs coupled with CD80 and 41BBL, respectively. The 19z1-CD80-41BBL is a 1st-generation CAR that combined with both CD80 and 41BBL

T cells require at least two distinct signals for full activation [115]. The first is delivered into T cells when TCR binds to its cognate antigenic peptides bound to MHC molecules on an antigen-presenting cell (APC). The second signal is provided when the costimulatory receptor on the T cell binds to its cognate ligand on the APC [115]. Antigenic stimulation without costimulation results in T cell anergy and unresponsiveness [116, 117]. CARs in which the antigen recognition domain is linked to the Fc receptor gamma chain (FcγR) or TCR zeta chain (CD3ζ) alone are known as “first-generation” CARs [118, 119]. Although the 1st-generation CARs exhibited cytotoxicity against target cells in-vitro and in-vivo, limited antitumor efficacy and poor persistence were observed in early-phase clinical trials [82, 120–122]. The insertion of a costimulatory unit into 1st-generation CARs, introduced as the second-generation CARs, enhanced T cell proliferation and in vivo persistence [122]. Third-generation CARs were also developed by fusing two costimulatory domains in a series. Most costimulatory molecules used in CARs belong to the Ig superfamily, such as CD28 and ICOS, or the TNF receptor superfamily (TNFRSF), such as 4-1BB, OX40, and CD27 [123]. A schematic picture of above-mentioned costimulatory molecules and their ligands has been illustrated in Fig. 4a.

Perhaps the best-characterized T cell costimulatory molecule is CD28, constitutively expressed on the surface of naïve T cells and some subsets of memory T cells [124]. It was first described in the 1980s as a co-receptor that enhanced TCR-induced proliferation and stimulated the differentiation of naive CD4 T cells [125, 126]. CD28, encoded by the CD28 gene located on human chromosome 2 (2q33), is a 44 kDa type I integral membrane glycoprotein that usually forms homodimers via disulfide bonds between cysteine residues positioned on the juxta transmembrane domain [125–127]. A single Ig-V-like domain on the extracellular portion of CD28 provides the structural specificity for interactions with its ligands, CD80 (B7–1) and CD86 (B7–2), expressed on APC upon activation [128]. CD28 contributes to proliferation [125, 129], IL-2 production [130, 131], survival [132–134], and metabolic activity [135] of naïve T cells by regulating the expression and activity of nuclear factor-κB (NF-κB), nuclear factor of activated T cells (NFAT), and activator protein 1 (AP-1) [136–138]. It is also involved in the cytoskeletal rearrangement, actin polymerization, and membrane rafts recruitment into the immune synapse, which maintains and boosts TCR-induced signaling [139–141].

The inducible T cell co-stimulator (ICOS) (CD278), the third member of the CD28/cytotoxic T lymphocyte-associated antigen-4 (CTLA-4) family, is expressed on activated T cells [123, 142]. ICOS interacts with another B7-related molecule, ICOSL, also known as B7H, B7RP-1, and GL-50, expressed by APCs. Although ICOS and CD28 are similar in structure and downstream pathways, they are not identical [123, 143]. The CD28/B7 engagement is required for the primary immune responses, whereas ICOS/B7RP-1 is essential for secondary immune responses [144, 145]. ICOS is crucial for the development and maintenance of human T helper 17 (Th17) cells [146] and also directs immunity towards humoral or inflammatory responses [144]. Although their function, both, is costimulatory for T-cell proliferation and cytokine secretion, the effects of ICOS on the costimulation of T-cells appear less potent than those exerted by CD28, probably because ICOS cross-linking does not induce IL-2 production [147].

The expression of 4-1BB (TNFRSF9, CD137, ILA), first discovered in the late 80 as an inducible costimulatory molecule on activated T cells, is mainly activation-induced and not restricted to T cells and detected on various types of non-T cells as well [148–150]. 4-1BB interaction with its ligand, 4-1BBL, can costimulate T cells by activating the NF-κB, c-Jun, and p38 downstream pathways independently of CD28 signals. In contrast to CD28, 4-1BB enhances T cell effector responses by stimulating proliferation, cytokine production, and cytolytic activity and inhibiting AICD of effector T cells, not naïve T cells [151, 152]. Furthermore, numerous studies have suggested that 4-1BB predominantly promotes CD8 T cell-mediated response [153, 154], and some also described a dual immunoregulatory function for 4-1BB. While it supports both CD4 and CD8 T cell-mediated responses in vitro, it preferentially augments clonal expansion and survival of CD8 T cells in vivo, suppressing CD4 + T-cell function [155, 156].

The expression of OX40 (TNFRSF4, CD134), first described as a T cell activation marker, can be induced on activated CD4 and CD8 T cells as well as on several other lymphoid and non-lymphoid cells. A slight expression can be noticed within the first hours of T cell activation in vitro and in vivo, peaking anywhere from 1 to 5 days after initial stimulation. Although TCR signals are sufficient for inducing OX40, the interaction of CD28 with its ligands can increase and maintain OX40 expression; T cell and APC-derived cytokines such as IL-1, IL-2, and TNF may also modulate its amount and length [157]. Its ligand, OX40L (TNFSF4), is not constitutively expressed but can be induced on APCs upon activation [157]. Known as the late costimulatory molecules of the TNFR family, 4-1BB and OX40 prolong T-cell persistence and promote the generation and survival of effector and memory T cells [158–160]. Hombach et al. demonstrated that in contrast with CD28-based CARs, OX40-costimulated CARs do not secrete IL-2 and IL-10. They also found that in a CD28-OX40 dual costimulatory CAR, OX40 represses IL-10 secretion induced by CD28 without affecting IL-2 and IFNγ production. This favorable feature of OX40 can be employed, particularly for treating solid tumors in which IL-10, an immunosuppressive cytokine, is secreted into the TME by tumor and stromal cells [161]. Concerning nanobody-based CAR-T cells, we observed that anti-HER2 nanobody-based CAR-T cells containing a combination of CD28-OX40 showed increased expansion level and cytotoxicity in vitro compared to CAR-T cells lacking OX40 [56]. Recently, Zhang et al. showed that OX40 signaling enhanced CAR-T cell survival through up-regulation of anti-apoptotic Bcl-2-like molecules and improved proliferation through increased activation of the NF-κB, MAPK, and PI3K-AKT pathways [162].

The CD27 (TNFRSF7) is constitutively expressed on T cells, NKT cells, NK cells, and other immune cells. Its expression on T cells is strongly upregulated following activation [158]. CD27 engagement by its ligand, CD70, supports CD28-mediated costimulation and the survival of proliferating T cells [163]. CD27/CD70 interaction can also augment the expansion and survival of effector cells and enhance the development of memory CD8 T cells. Likewise, it can promote proliferation, polarization, and cytokine production by CD4 T cells [164]. It has been reported that CD27-costimulated CARs exhibited an antitumor activity similar to 4-1BB or CD28-based CAR-T cells while providing a persistent comparable to that of 4-1BB-based CAR-T cells. The combination of CD27 and CD28 costimulatory domains in a 3rd-generation CAR produced encouraging clinical results in neuroblastoma, AML, and lymphoma patients [165].

Most clinical trials have used CD28 or 4-1BB-costimulated CARs to date [166]. Although T cells expressing CARs with either a 4-1BB or CD28 costimulatory domain have demonstrated similar antitumor activity, particularly against lymphomas [7, 10, 167, 168], T cells expressing CD28-costimulated CARs display higher cytokine production but lower persistence [111, 169–171]. The persistence of CAR costimulated by CD28 was identical to that achieved with CD3ζ alone, indicating that CD28 does not support human T-cell survival in vivo [172, 173]. The persistence of CD28-costimulated CAR-T cells can be improved by replacing CD28 with 4-1BB [174, 175] or CD27 [176] or by adding 4-1BB alone [175] or a combination of 4-1BB and CD27 [177]. CARs harboring the 4-1BB costimulatory domain mediate long-term survival of T cells in the circulation by maintaining central memory phenotype and relying on oxidative metabolism, whereas CD28-costimulated CARs promote effector memory differentiation and rely on aerobic glycolysis [50, 169, 178]. In efforts to find an ideal combination of CD28 and 4-1BB that would preserve the superior tumoricidal capacity of CD28-based CARs with the sustenance afforded by the 4-1BB-based CARs, Zhao et al. developed seven different structural configurations of CARs, three of which (1928z-41BBL, 19BBz-CD80, and 19z1-CD80-41BBL) coupled with costimulation ligands [179] (Fig. 4b). They found that the 1928z-41BBL and 19BBz-CD80 configurations showed more favorable properties regarding in vivo tumoricidal cytotoxicity, proliferation, persistence, and IRF7/IFNβ pathway induction. The 1928z-41BBL CAR, however, consistently outperformed the 19BBz-CD80 and emerged as the most potent configuration. They also observed that CD28 downregulation, occurring following activation, averts the activity of its constitutively expressed ligand (CD80) provided by the 19BBz-CD80 configuration [179]. Furthermore, the 19z1-CD80-41BBL configuration, in which CAR coupled with both CD80 and 41BBL simultaneously, was the least effective, expanding steadily but exerting inferior tumor control [179]. The lack of durable antitumor responses in CD28-costimulated CAR-T cells may also be due to tonic signaling and T cell exhaustion mediated by the CD28 costimulatory domain. Recently, Guedan et al. reduced T cell exhaustion, driven by CD28, and thus enhanced in vivo persistence of the CD28-based CARs targeting mesothelin by disrupting the interaction between the CD28 signaling domain and the SH2-domain of Grb2 via a single amino acid alteration [180]. CARs containing ICOS also showed better persistence when compared with CD28-based CARs. It is likely because ICOS activates PI3K signaling and, consequently, Akt signaling more potently than CD28. As shown by Guedan et al., expressing ICOS-based CAR in CD4 T cells not only improves the persistence of these cells but also enhances the in vivo persistence of CD8 T cells expressing either 4-1BB– or CD28-based CAR [111]. Despite accumulated data indicating poor persistence of CD28-based CARs, prolonged survival of anti-CD19 CARs containing CD28 costimulatory domains has been reported in more recent trials, suggesting that, aside from CD28, other elements incorporated into the CAR structure may also affect the CAR-T cells in vivo persistence [7, 79, 81, 111, 181, 182].

Different costimulatory domains can induce various downstream signaling pathways. Selecting a costimulatory unit or a combination that would heighten antitumor activity and maintain the long-term persistence of CAR-T cells is crucial. Besides costimulatory molecules discussed in this review, alternative costimulatory molecules, such as CD40 (TNFRSF5) [183], Herpes Virus Entry Mediator (HVEM) (TNFRSF14, CD270) [184], Glucocorticoid-Induced TNFR-Related protein (GITR) (TNFRSF18, CD357) [185], Myeloid Differentiation Primary Response 88 (MYD88)/CD40 [186], Toll-Like Receptor 2 (TLR2) [187], and Dectin-1, a C-type Lectin Receptor [188], have been explored to determine whether the incorporation of these molecules into CAR structure could improve clinical outcomes in hematological malignancies or heighten potential applications of CAR-T cell therapy in patients with solid tumors.

#### Activation domain

**Fig. 5:**
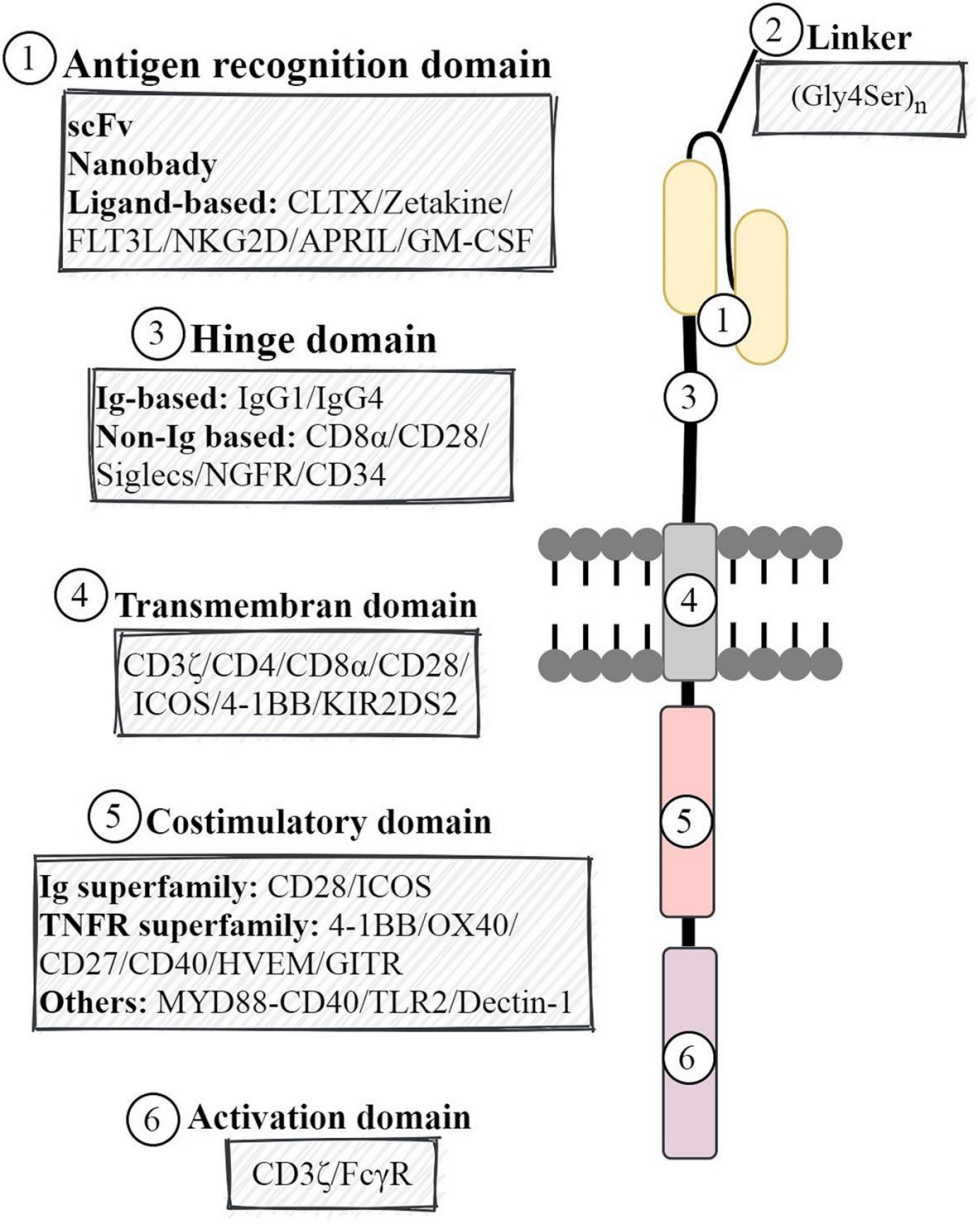
A schematic picture of all structural elements of CAR reviewed here

In the CAR construct, the activation motif is the critical component to trigger T cell activation signaling, including the initiation of cytotoxicity. The CD3ζ is the most common activation molecule used in CAR-T cells [189]; however, early studies also utilized the FcγR as the primary activating domain in CARs [118, 190]. All FDA-approved CARs have employed CD3ζ as the cytoplasmic activation domain (Fig. 1). The CD3ζ (CD247) is a component of the TCR complex comprising three tyrosine-rich sequences known as ITAMs, named ITAM1, ITAM2, and ITAM3, from the membrane-proximal to the membrane-distal direction. The ITAMs presented on the cytoplasmic region of CD3ζ are the phosphorylation sites recruiting the tyrosine-protein kinase ZAP70, which, in turn, triggers downstream signaling cascades. The two distal ITAMs (ITAM2 and ITAM3) display a lower binding affinity for ZAP-70 compared to ITAM1. In T cells, the quantity and diversity of ITAMs affect optimal signaling; however, Feucht et al. demonstrated that a single functional ITAM is sufficient for potent antitumor efficacy. CAR containing a single ITAM (either ITAM1, 2, or 3) outperformed the triple- and double-ITAM-containing CARs in vivo and limited T cell differentiation, resulting in an increased fraction of central memory CAR-T cells and increased persistence as well [191]. Fisher et al. designed a novel CAR construct in which an scFv targeting GD2 was linked to DAP10 as the sole signaling domain and transduced into γ/δ T cells. Due to the absence of the CD3ζ, these CAR-T cells depended on their native γ/δ TCR for activation. They could efficiently eradicate neuroblastoma cells; however, those target cells lacking γ/δ TCR ligands could escape [192]. A summary of all structural elements of CAR reviewed here has been shown in Fig. 5.

## Vector backbone

**Fig. 6:**
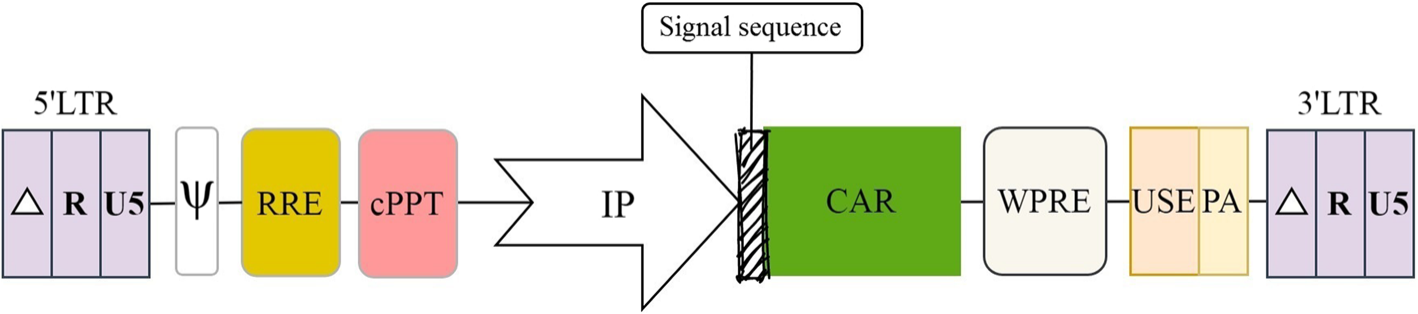
A schematic picture of a SIN LV vector backbone indicating where modular regulatory elements should be placed. LTR: Long Terminal Repeat, RRE: Rev-Response Element, cPPT: Central Polypurine Tract, IP: Internal Promoter, WPRE: Woodchuck Hepatitis Virus (WHV) Posttranscriptional Regulatory Element, USE: Upstream Sequence Element, PA: Polyadenylation Signal

Retroviral vectors are attractive tools for gene therapy due to the ability to integrate into the genome of target cells, a requisite for long-term expression, and maintaining a large cloning capacity, which is ideal for most clinical situations. In addition to the above-stated features common to all retroviral vectors, vectors derived from lentiviruses offer a unique advantage over their oncoretroviral counterparts: they can translocate across an intact nuclear membrane to transduce nondividing cells, the main potential targets of most gene therapies [193]. Lentiviral (LV) vectors used for gene therapy are predominantly derived from Human Immunodeficiency Virus type 1 (HIV-1). Two components are needed to make a virus-based gene delivery system: first, the packaging elements, encompassing the structural proteins and the enzymes required to generate an infectious particle, and second, the vector itself, that is, the genetic material transferred to the target cell [194]. The viral coding sequences are usually removed from the viral backbone and replaced by the gene of interest, such as the CAR. In this way, the transgene sequence is flanked by two long terminal repeats (LTRs), which are essential for the transgene integration into the host genome. The LTRs are identical in nucleotide sequence and organization and consist of U3-R-U5 regions in which the U3 is necessary for retroviral RNA transcription due to having endogenous enhancer/promoter sequences. Therefore, in wild-type retroviral-based vectors, transgene expression can be driven by the transcriptional sequences within the LTRs. However, to meet the safety requirements for clinical applications and minimize the risk of arising replication-competent recombinants (RCRs), self-inactivating (SIN) vectors are developed by removing a majority of the U3 region of the 3′ LTR. This deletion is transferred to the 5′ LTR of the proviral DNA during reverse transcription. Thus, SIN LV vectors retain all properties of their parent while lacking the ability to produce full-length vector RNA in transduced cells [193]. SIN LV vectors are the gold standard for CAR-T cell production due to the highest transduction/transfection rate. However, the efficiency of gene delivery by these vectors relies on some elements that should be present in the vector backbone or expression cassette (Fig. 6).

### Promoter

A gene transfer approach must be effectively directed to the specific tissues/cells where is desired, and the resulting transgene expression should be at a level required for a specific application [195]. Regarding CAR-T cell therapy, the CAR density on the T cell surface must be high enough to recognize the target antigen but not too high to trigger antigen-independent tonic signaling or drive off-tumor on-target cytotoxicity as a result of inappropriate recognition of target antigen on cells rather than tumor cells. Lentiviral vectors can transfer CAR construct into activated CD4 and CD8 human T cells with high efficiency, but its expression level depends on the promoter that drives its transcription. The U3 region of the 5′ LTR possesses endogenous enhancer/promoter sequences; however, it is removed in SIN LV vectors to enhance safety. Accordingly, for transgene expression in SIN vectors, internal promoters are employed. Selecting an appropriate promoter is a fundamental step toward a successful CAR-T cell therapy [196]. Promoter is a cis-acting element within the vector backbone that can dictate the level and duration of transgene expression and restrict expression to the specific tissues/cells, two critical goals desired for clinical applications. Various viral promoters (immediate-early cytomegalovirus [CMV], murine stem cell virus [MSCV] or spleen focus forming virus [SFFV] promoter), and cellular promoters (human elongation factor 1α-subunit [EF1α], human phosphoglycerate kinase1 [PGK], human ubiquitin C [UbiC] or the chicken β-actin and its derivative CAG) have been evaluated for transgene expression in lentiviral vectors. CMV and EF1α are the most commonly used promoters for CAR expression in T cells.

During productive infection of Human CMV (HCMV)*,* viral genes are expressed, from immediate-early (IE) to early (E) and late (L) genes, respectively, in a temporal cascade [197]. The expression of IE genes is driven by a potent enhancer-containing promoter. This Major IE Enhancer and Promoter (MIEP) is active in various cell types and is the most commonly used promoter in mammalian expression plasmids [197]. Successful gene therapy with CAR-expressing T cells relies on the ability of T cells to maintain adequate receptor expression for a long time. The EF1a promoter is a strong promoter frequently used for CAR expression, as it often drives a strong and stable expression regardless of tissue specificity or the T cell activation state [169, 198]. Milone et al. evaluated several promoters, including EF1α, CMV, PGK, and UbiC, to identify the one that can drive the highest stable expression of a transgene in primary CD4 and CD8 T cells [169]. They found that although the CMV promoter caused a high expression level of the green fluorescent protein (GFP) early after transduction, expression dropped to < 25% of the initial expression level after 10 days of culture. In contrast, the EF1α promoter induced the highest level of GFP expression and optimally maintained it in both CD4 and CD8 T cells [169]. The sustained proliferation of CAR-T cells depends on CAR structure and high expression, the latter of which is required but not sufficient. When CARs having the CD28 transmembrane and cytosolic domain is expressed under the control of the EF1α promoter, they display a constitutive growth phenotype. Despite the constitutive growth phenotype, these CARs showed inferior antitumor effects and engraftment in vivo [199]. That is probably the explanation for why the two FDA-approved CD28-based CAR products, including Yescarta (axicabtagene ciloleucel, Kite Pharma Inc.) and Tecartus (brexucabtagene autoleucel, Kite Pharma Inc.) use the MSCV promoter instead of EF1α for CAR expression. The retroviral vectors incorporating MSCV LTR have been widely used in pre-clinical and clinical studies to drive high-level and long-term maintenance of CAR expression [200, 201].

Since heterogeneous expression of the CAR makes it challenging to ensure consistent behavior among individual CAR-T cells as their avidity toward the antigen can vary, targeted insertion of CAR into the first exon of the constant chain of the TCRα gene (TRAC) allows for a homogenous and consistent TCR-like expression, thus dodging issues related to the variegated transgene expression [202]. Eyquem et al. used the CRISPR/Cas9 paired with an adeno-associated virus (AAV) vector repair matrix to insert a CD19-specific CAR construct into the TRAC locus. CAR-T cells generated by CRISPR knock-in strategy outperformed the CAR-T cells generated via retroviral infection, both in vitro and in vivo [203]. These studies also demonstrated that targeting the CAR into the TRAC locus results in receptor internalization and re-expression cycle that is much more closely matched to the normal receptor, ultimately leading to a more sustained and effective antitumor response. Furthermore, this elegant CAR-knock-in and TCR-knockout strategy not only averts tonic CAR signaling by precisely regulating the expression of the CAR but also minimizes the risk of graft versus host disease (GVHD) by diminishing the expression of αβ TCRs on the T cell surface [203].

The expression of multiple genes requires self-cleaving 2A-like peptides of the Foot and Mouth Disease Virus (FMDV) [204], an internal ribosome entry site (IRES) [205], or the use of several promoters. The two latter strategies are the most widely used. It has been shown that the expression levels of the genes upstream and downstream of the IRES element vary, with the downstream gene typically expressed at lower levels [206]. Using two separate, divergent promoters may significantly increase the size of expression cassettes, and also probable different tissue specificity and mutual interference between them may prevent efficient co-expression in the same target cell [207, 208]. However, an alternative approach is to employ a single, compact bidirectional promoter [209]. He et al. could successfully use a bidirectional promoter to express dual CAR cassettes in the Sleeping Beauty system; however, they found that the investigated bidirectional promoters are sub-optimal for lentiviral production of long RNA encoding long dual-CAR constructs [210]. EF1α can be the best choice for driving lentiviral-based vectors containing a long RNA in CAR-T cells. Hosseini Rad et al. evaluated four strong well-characterized promoters, including EF1α, CMV, hPGK, and RPBSA, for optimal expression of a long and complicated RNA encoding multiple gene products in CAR-T cells and found that EF1α is the best choice for driving short as well as long RNA in CAR-T cells [196]. They also discovered that the EF1α promoter exhibited the best transduction efficiency, killing ability, and cytokine production. Furthermore, the authors observed a reduction in CAR expression driven by the hPGK and RPBSA promoters, which retained acceptable killing ability but reduced cytokine production [196].

### Signal peptide

Recognition of target antigen typically requires CAR expression on the T cell surface. CAR protein is a type I membrane protein that must be trafficked through the secretory pathway to the plasma membrane, where it can be anchored to and exert its function. Accordingly, the CAR coding sequence starts with a signal peptide (SP). The SP, also known as the leading peptide, was first described in 1975 as a short transient peptide, predominantly found at the N-terminal of secretory and type I membrane proteins that direct the nascent polypeptide chain to the endoplasmic reticulum (ER) membrane [211, 212]. The SPs typically contain 25-30aa; however, longer SPs (up to 140aa) are also seen in eukaryotes; they are predominantly organelle-targeting, which remain stable even after protein maturation, and usually add extra functions to the protein targeting [213]. The SPs act like address tags and mediate the translocation of secretory proteins across intracellular membranes and final localization. They are more precisely required for protein translocation across the first membrane on the secretory pathway and thus universally control the entry of all proteins to the secretory pathway [211]. Although SPs may vary in length and sequence, they are found in both prokaryotes and eukaryotes and share a conserved tripartite structure, commonly characterized by a positively charged N-terminal region, a 9-to 12-residue-long hydrophobic stretch in the center that forms an α-helical conformation, and a polar C-terminal region with the cleavage site for signal peptidase [212]. As a nascent protein emerges from the ribosome, the signal peptide is recognized by the signal recognition particle (SRP), a cytoplasmic ribonucleoprotein [214]. SRP pauses elongation of the nascent polypeptide chain until the SRP–SP–ribosome complex interacts with an SRP receptor on the ER membrane. Upon interaction, the nascent chain is inserted into the ER translocon, and polypeptide chain elongation resumes. The SP is then cleaved off by a signal peptidase residing in the ER while the growing protein passes through the ER membrane [215, 216]. The SPs usually incorporated into the CARs are derived from human CD8α [217], IL-2 [218], GM-CSF receptor (GM-CSF) α chain [219], or murine Ig-kappa (IgK) [220]. Wang et al. chose the leader sequence of GM-CSFRα at the beginning of a CD19-specific CAR and a truncated form of human epidermal growth factor receptor (huEGFRt) coding sequences, based on its capacity to sort type I transmembrane proteins to the plasma membrane in T cells [221]. Recently, Ping et al. designed CAR-T cells that could secret α-PD-1 scFv in solid tumors. They compared six signal peptides frequently used in engineering secretory proteins, including Secrecon, murine IgK V-III region (IgKVIII), human IgKVIII, CD33, human tissue plasminogen activator (TPA), a consensus 16aa signal peptide, and native secreted alkaline phosphatase (SEAP) [222], to find the one that can enhance extracellular accumulations of anti-PD-1 scFv. They observed that the one derived from human IgK VIII was the best choice as the secreting capacity of anti-PD-1 scFv was significantly enhanced by this leading peptide [223].

### RRE

Retroviruses such as lentiviruses employ various mechanisms to regulate the expression of alternatively spliced viral mRNAs. The presence of suboptimal splice sites allows for differential expression of several mRNAs from a single pre-RNA. Lentiviruses, including HIV-1, utilize Rev/Rex proteins that act in trans to regulate the nuclear export of unspliced or singly-spliced mRNAs required for the expression of structural and enzymatic proteins and progeny viral RNA genomes as well [224]. After integration into the host genome, during the early phase of HIV-1 life, the viral DNA called the provirus now on, is acted upon by cellular transcription factors to express viral genes. The early population of the transcripts is a fully-spliced mRNA (2 kb) exported to the cytoplasm to be translated into viral regulatory proteins, including Tat, Nef, and Rev. The Rev is a nucleocytoplasmic shuttle protein necessary for virus replication. Rev, later in the viral life cycle, is imported to the nucleus to mediate the export of the Rev Response Element (RRE)-containing unspliced (9 kb) or partially spliced (4 kb) mRNAs to the cytoplasm, where they are translated into viral proteins, such as Gag, Gag-Pol, Env, and accessory proteins, or packaged as the viral genome into newly budding virions [225]. The interaction between Rev and RRE is necessary for exporting such intron-containing mRNAs, which are usually kept in the nucleus to be spliced or degraded. The RRE is a cis-acting element located at the junction between the SU (gp120) and TM (gp41) domains of the ENV gene on viral genomic RNA [226], with a well-conserved sequence (351 nucleotides) and highly branched structure (an approximately equimolar mixture of 4 and 5 stem-loop conformations) [227] that provides an architectural scaffold with a high affinity for Rev binding. DiMattia et al., using X-ray crystallography and cryo-EM, described an “A”-shaped architecture for RRE, which allows 8–12 Rev-subunits to bind to and mediate nuclear export [228]. Both Rev oligomerization and its interaction with RRE, initiated by binding the Rev arginine-rich motif (ARM) to a high-affinity Rev-binding site known as stem-loop IIB on the RRE, are critical for viral RNAs export and, consequently, virus replication [229]. After assembly of the Rev-RRE ribonucleoprotein (RNP) complex, the nuclear export sequences (NESs), displayed on the Rev, interact with the host Crm1/RanGTP nuclear-export machinery and facilitate the nuclear export of viral intron-containing mRNAs [225, 230, 231].

### cPPT

To be inserted into the host genome, the single-stranded RNA genome needs to be converted into double-stranded DNA before nuclear import. While synthesis of the first (minus) strand of DNA is initiated by a cellular tRNA molecule already packaged into the retroviral particles, synthesis of the plus-strand DNA is primed by a short purine-rich remnant of the viral RNA known as the polypurine tract (PPT), selectively preserved when the RT digest the viral RNA from the nascent RNA/DNA hybrid [232]. Besides this copy of PPT, shared by all retroviruses, HIV-1 carries a second copy of the PPT, known as cPPT, placed near the center of the genome in the integrase open reading frame [233]. Thus, synthesis of the plus-strand DNA in HIV-1 is primed by both PPT and cPPT, resulting in two discrete plus-strand segments, each covering half of the viral genome [234]. Synthesis of upstream plus-strand DNA initiated at the PPT continues for approximately 99 nucleotides downstream of cPPT, expelling the centrally initiated downstream plus-strand DNA, and is then stopped at the central termination sequence (CTS), creating a triple-stranded DNA structure at the cPPT. The 99-nucleotide-long overlapping DNA, also known as the central DNA flap, is vital for the pre-integration complex (PIC) formation and thus nuclear import [235]. Defects in the DNA flap formation result in the trapping of the PIC at the cytoplasmic side of the nuclear pore, prohibiting nuclear entry of the HIV-1 genome [236]. It has been shown that the insertion of a 118-bp sequence of the HIV-1 containing the cPPT/CTS element into lentiviral vectors enhances transduction efficiency, up to 85% in T cells pre-activated with IL-2 and PHA, by facilitating the nuclear import of transgene through a central DNA flap [237, 238]. Also, studies demonstrated that the presence of the cPPT/CST in lentiviral vectors leads to a 10-fold increase in the amount of integrated DNA and a 5- to 10-fold increase in the transduction efficiency, differing based on cell type [239]. Studies also revealed that initiating plus-strand synthesis at two separate sites in HIV-1 derived vectors would decrease the time during which minus-strand viral DNA remains single-stranded, thus averting its exposure to cellular enzymes, particularly cytidine deaminases of the APOBEC family known as natural defense barriers against HIV infection, that act on newly synthesized single-stranded viral DNA [232].

### Poly(a) signal and USE

A critical step in the mRNA 3′-end processing is polyadenylation, the consequences of which may impact mRNA stability and translation efficiency. Like eukaryotes, retroviruses need two cis-acting elements (together known as the core polyA site) for transcription termination and polyadenylation, including an almost invariant AAUAAA sequence, placed 10–30 nucleotides upstream of the cleavage site, and a more variant GU/U-rich sequence positioned immediately downstream [240]. In some retroviruses, the AAUAAA sequence is placed in the U3 region, while in all lentiviruses, including HIV-1, it is present in the R region [241].

HIV-1, in common with all retroviruses, has two LTRs (U3-R-U5), which are identical in nucleotide sequence and organization. Transcription starts at the junction of U3-R, and polyadenylation occurs at the junction of R-U5. Therefore, it is expected that the transcript initiated at + 1 nucleotide in the 5′ LTR would be polyadenylated at the end of the R sequence in the 5′ LTR itself, resulting in a truncated viral RNA [240]. To prevent premature termination and polyadenylation, the promoter-proximal polyA site in the 5′ LTR must be suppressed, while the promoter-distal polyA site in the 3′ LTR has to be selectively and efficiently used to polyadenylate all resulting viral RNAs [241]. Therefore, lentiviruses have evolved with additional sequences to support and ensure efficient transcriptional termination and prevent the possibility of read-through into cellular genes. In SIN vectors, most of the U3 region is deleted from LTRs to enhance safety. However, in the case of HIV, this deletion results in leaky polyadenylation suggesting that U3 contains termination enhancer motifs or USEs. These USEs are U-rich sequences placed upstream of the AAUAAA sequence serving a critical role in selecting the 3′ polyA site in preference to an identical polyA site at the 5′ end of mRNA [242, 243]. Efficient 3′-end processing of HIV-1 has been shown to depend on a stem-loop structure that places the USE and the core polyA site close together [244].

Schambach et al. incorporated seven different USEs derived from various viral or cellular genes into SIN LV vectors to find the best one for improving viral titer and gene expression. They observed that the USE derived from simian virus 40 (SV40) late mRNA, especially when duplicated (2xSV), provided the best results, improving viral titer (up to threefold) and gene expression (by 45–100%) [244]. Interestingly, the relatively small 2xSV USE (100 bp) was nearly as potent as the WPRE (600 bp) in enhancing viral titer and transgene expression. However, the level of the effects depended on other elements, such as promoter and target cell type. They also found that the 2xSV USE was superior to the WPRE in suppressing transcriptional read-through, thus improving vector efficiency and biosafety [244]. Hager et al. investigated whether the inclusion of a strong polyA signal as part of an internal transcription unit has the potential to overcome problems regarding insufficient termination. They found that an internal polyA signal increases transgene expression to levels comparable to that typically observed in the presence of the WPRE but decreases viral titer in a promoter-dependent manner [245]. Studies have shown that an expression cassette containing a combination of WPRE (a shortened version with 247 bp), an SV40 late USE, and a full-length SV40 late polyA signal drives the highest levels of gene expression compared to vectors containing each element alone [246] (Fig. 6).

### WPRE

**Fig. 7:**
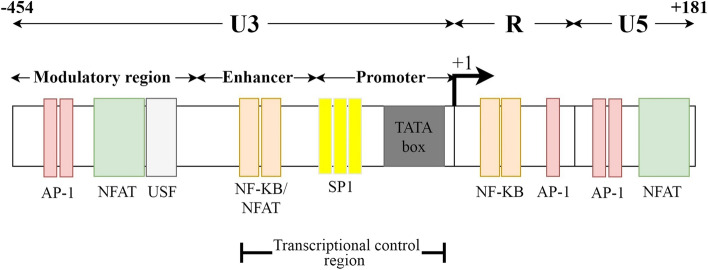
A schematic picture of the LTR and also termination signals present within the U3 region. Studies showed that besides USE, two additional elements in U3, the transcriptional control region and the nuclear factor of activated T cells/upstream stimulatory factor (NFAT/USF) binding region, contribute significantly to lentiviral LTR transcriptional termination

The WPRE, a post-transcriptional regulatory element (PRE) of the woodchuck hepatitis virus (WHV), is primarily known as an RNA export element that facilitates the accumulation of surface antigen transcripts in the cytoplasm from the intronless hepadnavirus genome [247]. WPRE can substantially enhance viral titers and transgene expression from various RNA and DNA viral vectors [248–250] when placed in the sense orientation in the 3′-untranslated region (3′-UTR), upstream of the polyadenylation signal [251]. Xu et al. observed that the insertion of WPRE in viral vectors could increase the expression of the reporter gene up to 7-fold in vitro and up to 50-fold in vivo [250]. The positive impact of the WPRE on gene expression is not due to an enhanced rate of transcription, viral mRNA half-life, or nuclear export; it instead acts post-transcriptionally and increases the efficiency of mRNA 3′-end processing [248, 251]. Studies demonstrated that oncoretroviruses, but not lentiviruses, usually display a high transcriptional readthrough activity in the 3′ LTR [252]. This reduced 3′ termination efficiency may cause RNA instability during transgene expression and also raise safety issues regarding the increased risk of activating or capturing downstream cellular oncogenes [252]. SIN LV vectors, which lack the U3 region, also suffer from a leaky transcription termination and exhibit high transcriptional readthrough activity, suggesting that additional termination signals must be present within the U3 sequence [253]. Further studies showed that besides USE, two additional elements in U3, including the transcriptional control region and the nuclear factor of activated T cells/upstream stimulatory factor (NFAT/USF) binding region, contribute significantly to lentiviral LTR transcriptional termination (Fig. 7). Restoration of the transcriptional control region alone reduces readthrough by 70–80%, while insertion of the NFAT/USF binding region reduces RNA readthrough to a level even lower than that of the wild type LTR [254]. However, instead of restoring the U3, which causes safety issues, genetic elements, such as WPRE, can be inserted into SIN LV vectors to reduce readthrough without causing any adverse effects.

Like the WPRE, PRE found in the human hepatitis B virus (HBV) (HPRE) can increase the level of gene expression; however, studies showed that WPRE displays two to three times more potent activity [247]. WPRE (600 bps) and HPRE (553 bp) have two homologous subelements, called α and β, while WPRE contains an additional subelement named gamma [247]. Both WPRE and HPRE contain AU-rich motifs in the β-loop that resemble the USE core sequence. WPRE also possesses a second USE core sequence-like motif in the γ-loop, which may explain its potency of transcript termination and increased total viral mRNA over HPRE [251]. However, Choi et al. discovered that the insertion of a shortened WPRE sequence (247 bp), containing approximately 41.2% of the original size, into adeno-associated virus (AAV) vectors causes a level of transgene expression comparable to that of wild-type WPRE while providing more space for larger transgenes [246].

## Conclusion and future perspectives

The optimal design of a CAR construct requires a comprehensive understanding of the features of each component alone and in combination with others within the CAR. As CAR constructs become more complex and more elements come into play, a deep understanding of the impact of distinct domains would likely improve the rational design of CAR-T cells to fit the specific needs of individual patients. However, even an optimized CAR may not overcome all the hindrances presented by the complex nature of tumors. Furthermore, viral vectors, particularly lentiviral vectors, may be highly efficient in CAR-T cell production, but several critical features discourage their use in the clinic, supporting nonviral approaches [255]. The possibility of insertional mutagenesis, caused by the random integration of viral DNA into the host genome, the limited cargo size capacity, and the high manufacturing complexity and cost are some drawbacks associated with viral vectors, prompting researchers to look for other alternatives. Transposable elements (transposons) are the most common alternatives to viral vectors with a vast potential for diverse applications in genetic engineering, including CAR-T cell therapies, since they are easier and less expensive to manufacture due to their plasmid-based nature and offer a larger cargo size capacity to deliver multiple transgenes. Various transposon-based systems, including the Sleeping Beauty (SB) [256], the piggyBac (PB) [257], and Tol2 transposon [258], have been reported for CAR-T cell production as these systems provide safe and reliable DNA transfer into T-cells. Like viral-based CAR-T cells, transposon-mediated CAR-T cell clinical applications mainly focus on blood malignancies and target the CD19 antigen [259–262]. Kebriaei et al. (2016) reported the first human application of the SB system for 26 patients with advanced NHL or ALL. All patients received a single dose of patient- or donor-derived CD19-specific CAR-T cells generated with SB in the phase I adjuvant setting following autologous (NCT00968760) or allogeneic (NCT01497184) hematopoietic stem cell transplantation (HSCT). They utilized high-throughput sequencing to analyze the CAR integration patterns in T cells, genetically modified by SB transposon/transposase plasmids, and observed that integrations were widely distributed throughout the genome with no bias [259]. For patients who received CAR-T cells after autologous HSCT (*n* = 7), the 30-month progression-free survival (PFS) and overall survival (OS) rates were around 83 and 100%, respectively. For those who received CAR-T cells after allogeneic HSCT (*n* = 19), the respective 12-month rates were 53 and 63% [259]. Recently, Li et al. used piggyBac-produced CD19-specific CAR-T cell therapy for a male patient of triple-hit R/R DLBCL with TP53 mutation (ChiCTR1800018111). Triple-hit lymphoma (THL), a relatively rare subset of DLBCL identified in approximately 1% of DLBCL cases, carries concurrent MYC, BCL2, and BCL6 rearrangements. Despite grade 2 cytokine release syndrome, the patient achieved CR two-month after CAR infusion and has still kept sustained CR for over 24 months [262]. Recent studies on transposon-mediated CAR-T cell therapy have mainly focused on two areas: 1) developing novel transposon systems to deliver multiple transgenes, such as the hyperactive Tc Buster, which has been originally isolated from the red flour beetle and shown comparable transposition efficiency to the SB and PB [263], 2) designing novel platforms to generate universal allogeneic CAR-T cells, such as the CRISPR-Cas9 ribonucleoparticles (RNP)-minicircles (mc) SB transposon platform optimized by Tipanee et al. to express CD19-specific CAR while inactivating allogeneic TCRs [264]. Although viral and nonviral techniques have their benefits and drawbacks, the question that needs to be answered is whether nonviral methods will hold the potential to meet these challenges and take CAR technology beyond the hematological malignancies.

## Data Availability

Not applicable.

## Abbreviations

CAR: Chimeric Antigen Receptor
FDA: Food and Drug Administration
MHC: Major Histocompatibility Complex
scFv: Single-Chain Variable Fragment
CR: Complete Remission
TSA: Tumor-Specific Antigen
TAA: Tumor-Associated Antigen
MM: Malignant Mesothelioma
GBM: Glioblastoma Multiforme
TanCAR: Tandem CAR
iCAR: Inhibitory CAR
PSMA: Prostate-Specific Membrane Antigen
R/R: Relapsed/Refractory
NHL: Non-Hodgkin Lymphoma
ALL: Acute Lymphoblastic Leukemia
LBCL: Large B-cell Lymphoma
PD-1: Programmed Cell Death Protein-1
CTLA-4: Cytotoxic T-Lymphocyte-Associated Antigen 4
HcAb: Heavy-chain-only Antibodies
MUC1: Mucin 1
CLTX: Chlorotoxin
AML: Acute Myeloid Leukemia
LSCs: Leukemic Stem Cells
FLT3L: FMS-like Tyrosine Kinase 3 Ligand
NKG2D: Natural Killer Group 2D
APRIL: A Proliferation-Inducing Ligand
TNFSF13: Tumor Necrosis Factor Ligand Superfamily Member 13
BCMA: B-cell Maturation Antigen
TACI: Transmembrane Activator and Calcium-Modulator and Cyclophilin Ligand
GM-CSF: Granulocyte-Macrophage Colony-Stimulating Factor
JMML: Juvenile Myelomonocytic Leukemia
Gly: Glycine
Ser: Serine
V-domain: Variable domain
Penn: University of Pennsylvania
CHOP: Children’s Hospital of Philadelphia
NCI: National Cancer Institute
IS: Immune Synapse
HD: Hinge Domain
ROR1: Receptor-Tyrosine Kinase-Like Orphan Receptor 1
FcγRs: Fc-gamma Receptors
DC: Dendritic Cells
NK cells: Natural Killer Cells
ADCC: Antibody-Dependent Cell-Mediated Cytotoxicity
AICD: Activation-Induced T-Cell Death
IVIG: Intravenous Immunoglobulin
TM: Transmembrane
TMD: Transmembrane Domain
LAG-3: Lymphocyte Activation Gene-3
CRS: Cytokine Release Syndrome
Siglecs: Sialic Acid-Binding Immunoglobulin-Type Lectins
NGFR: Nerve Growth Factor Receptor
ICD: Intracellular Domain
KIR: Killer Immunoglobulin-Like Receptor
ITAM: Immunoreceptor Tyrosine-Based Activation Motif
TCR: T-cell Receptor
APC: Antigen-Presenting Cell
CD3ζ: TCR Zeta Chain
TNF: Tumor Necrosis Factor
TNFRSF: TNF Receptor Superfamily
NF-κB: Nuclear Factor-κB
NFAT: Nuclear Factor of Activated T Cells
AP-1: Activator Protein 1
ICOS: Inducible T-Cell Co-Stimulator
Th17: T Helper 17
TME: Tumor Microenvironment
NKT cells: Natural Killer T Cells
HVEM: Herpes Virus Entry Mediator
GITR: Glucocorticoid-Induced TNFR-Related Protein
MYD88: Myeloid Differentiation Primary Response 88
TLR2: Toll-Like Receptor 2
LV: Lentiviral
HIV-1: Human Immunodeficiency Virus Type 1
LTRs: Long Terminal Repeats
RCRs: Replication-Competent Recombinants
SIN vectors: Self-Inactivating Vectors
CMV: Cytomegalovirus
MSCV: Murine Stem Cell Virus
SFFV: Spleen Focus Forming Virus
EF-1α: Elongation Factor 1α-Subunit
PGK: Phosphoglycerate Kinase
UbiC: Ubiquitin C
IE: Immediate-Early
E: Early
L: Late
MIEP: Major IE Enhancer and Promoter
GFP: Green Fluorescent Protein
TRAC: TCRα Subunit Constant Gene
AAV: Adeno-Associated Virus
GVHD: Graft Versus Host Disease
FMDV: Foot and Mouth Disease Virus
IRES: Internal Ribosome Entry Site
SP: Signal Peptide
ER: Endoplasmic Reticulum
SRP: Signal Recognition Particle
IgK: Ig-Kappa
huEGFRt: Human Epidermal Growth Factor Receptor
TPA: Human Tissue Plasminogen Activator
SEAP: Secreted Alkaline Phosphatase
RRE: Rev Response Element
Cryo-EM: Cryogenic Electron Microscopy
ARM: Arginine-Rich Motif
RNP: Ribonucleoprotein
NESs: Nuclear Export Sequences
RT: Reverse Transcriptase
PPT: Polypurine Tract
cPPT: Central Polypurine Tract
CTS: Central Termination Sequence
PIC: Pre-Integration Complex
APOBEC family: Apolipoprotein B mRNA Editing Catalytic Polypeptide-like family
USEs: Upstream Enhancer Elements
SV40: Simian Virus 40
WPRE: Post-Transcriptional Regulatory Element (PRE) of the Woodchuck Hepatitis Virus (WHV)
UTR: Untranslated Region
NFAT/USF: Nuclear Factor of Activated T Cells/Upstream Stimulatory Factor
HBV: Human Hepatitis B Virus
HPRE: Post-Transcriptional Regulatory Element of Human Hepatitis B Virus
SB: Sleeping Beauty
PB: PiggyBac
HSCT: Hematopoietic Stem Cell Transplantation
PFS: Progression-Free Survival
OS: Overall Survival
DLBCL: Diffuse Large B-cell Lymphoma
THL: Triple-hit Lymphoma
RNP: Ribonucleoparticles
mc: Minicircles

## References

[1] US Food & Drug Administration (2018). FDA approves tisagenlecleucel for adults with relapsed or refractory large B cell lymphoma.

[2] US Food & Drug Administration (2017). FDA approves axicabtagene ciloleucel for large B-cell lymphoma.

[3] US Food & Drug Administration (2020). FDA approves brexucabtagene autoleucel for relapsed or refractory mantle cell lymphoma.

[4] US Food & Drug Administration. FDA approves lisocabtagene maraleucel for second-line treatment of large B-cell lymphoma. https://www.fda.gov/drugs/resources-information-approved-drugs/fda-approves-lisocabtagene-maraleucel-second-line-treatment-large-b-cell-lymphoma. 2022.

[5] US Food & Drug Administration (2021). FDA approves idecabtagene vicleucel for multiple myeloma.

[6] US Food & Drug Administration (2022). FDA approves ciltacabtagene autoleucel for relapsed or refractory multiple myeloma.

[7] Neelapu SS, Locke FL, Bartlett NL, Lekakis LJ, Miklos DB, Jacobson CA (2017). Axicabtagene Ciloleucel CAR T-cell therapy in refractory large B-cell lymphoma. N Engl J Med.

[8] Maude SL, Laetsch TW, Buechner J, Rives S, Boyer M, Bittencourt H (2018). Tisagenlecleucel in children and young adults with B-cell lymphoblastic leukemia. N Engl J Med.

[9] Schuster SJ, Svoboda J, Chong EA, Nasta SD, Mato AR, Anak Ö (2017). Chimeric antigen receptor T cells in refractory B-cell lymphomas. N Engl J Med.

[10] Park JH, Rivière I, Gonen M, Wang X, Sénéchal B, Curran KJ (2018). Long-term follow-up of CD19 CAR therapy in acute lymphoblastic leukemia. N Engl J Med.

[11] Fry TJ, Shah NN, Orentas RJ, Stetler-Stevenson M, Yuan CM, Ramakrishna S (2018). CD22-targeted CAR T cells induce remission in B-ALL that is naive or resistant to CD19-targeted CAR immunotherapy. Nat Med.

[12] Brudno JN, Kochenderfer JN (2016). Toxicities of chimeric antigen receptor T cells: recognition and management. Blood.

[13] Sterner RC, Sterner RM (2021). CAR-T cell therapy: current limitations and potential strategies. Blood Cancer J.

[14] Glockshuber R, Malia M, Pfitzinger I, Plückthun A (1990). A comparison of strategies to stabilize immunoglobulin Fv-fragments. Biochemistry.

[15] van der Stegen SJ, Hamieh M, Sadelain M (2015). The pharmacology of second-generation chimeric antigen receptors. Nat Rev Drug Discov.

[16] Ramakrishna S, Highfill SL, Walsh Z, Nguyen SM, Lei H, Shern JF (2019). Modulation of target antigen density improves CAR T-cell functionality and persistence. Clin Cancer Res.

[17] Watanabe K, Terakura S, Martens AC, van Meerten T, Uchiyama S, Imai M (2015). Target antigen density governs the efficacy of anti-CD20-CD28-CD3 ζ chimeric antigen receptor-modified effector CD8+ T cells. J Immunol.

[18] Caruso HG, Hurton LV, Najjar A, Rushworth D, Ang S, Olivares S (2015). Tuning sensitivity of CAR to EGFR density limits recognition of Normal tissue while maintaining potent antitumor activity. Cancer Res.

[19] Liu X, Jiang S, Fang C, Yang S, Olalere D, Pequignot EC (2015). Affinity-tuned ErbB2 or EGFR chimeric antigen receptor T cells exhibit an increased therapeutic index against tumors in mice. Cancer Res.

[20] Olson ML, Mause ERV, Radhakrishnan SV, Brody JD, Rapoport AP, Welm AL, et al. Low-affinity CAR T cells exhibit reduced trogocytosis, preventing rapid antigen loss, and increasing CAR T cell expansion. Leukemia. 2022;36(7):1943-6. 10.1038/s41375-022-01585-2. 10.1038/s41375-022-01585-2 PMC9252916 35490197

[21] Ghorashian S, Kramer AM, Onuoha S, Wright G, Bartram J, Richardson R (2019). Enhanced CAR T cell expansion and prolonged persistence in pediatric patients with ALL treated with a low-affinity CD19 CAR. Nat Med.

[22] Drent E, Themeli M, Poels R, de Jong-Korlaar R, Yuan H, de Bruijn J (2017). A rational strategy for reducing on-target off-tumor effects of CD38-chimeric antigen receptors by affinity optimization. Mol Ther.

[23] Porter DL, Levine BL, Kalos M, Bagg A, June CH (2011). Chimeric antigen receptor-modified T cells in chronic lymphoid leukemia. N Engl J Med.

[24] Han L, Zhang J-S, Zhou J, Zhou K-S, Xu B-L, Li L-L (2021). Single VHH-directed BCMA CAR-T cells cause remission of relapsed/refractory multiple myeloma. Leukemia.

[25] Wagner DL, Fritsche E, Pulsipher MA, Ahmed N, Hamieh M, Hegde M (2021). Immunogenicity of CAR T cells in cancer therapy. Nat Rev Clin Oncol.

[26] Xu J, Chen L-J, Yang S-S, Sun Y, Wu W, Liu Y-F (2019). Exploratory trial of a biepitopic CAR T-targeting B cell maturation antigen in relapsed/refractory multiple myeloma. Proc Natl Acad Sci.

[27] Cao J, Wang G, Cheng H, Wei C, Qi K, Sang W (2018). Potent anti-leukemia activities of humanized CD19-targeted chimeric antigen receptor T (CAR-T) cells in patients with relapsed/refractory acute lymphoblastic leukemia. Am J Hematol.

[28] Turtle CJ, Hanafi LA, Berger C, Hudecek M, Pender B, Robinson E (2016). Immunotherapy of non-Hodgkin's lymphoma with a defined ratio of CD8+ and CD4+ CD19-specific chimeric antigen receptor-modified T cells. Sci Transl Med.

[29] Gorovits B, Koren E (2019). Immunogenicity of chimeric antigen receptor T-cell therapeutics. BioDrugs.

[30] Han X, Wang Y, Wei J, Han W (2019). Multi-antigen-targeted chimeric antigen receptor T cells for cancer therapy. J Hematol Oncol.

[31] Wei J, Han X, Bo J, Han W (2019). Target selection for CAR-T therapy. J Hematol Oncol.

[32] Kalos M, Levine BL, Porter DL, Katz S, Grupp SA, Bagg A (2011). T cells with chimeric antigen receptors have potent antitumor effects and can establish memory in patients with advanced leukemia. Sci Transl Med.

[33] Wang D, Starr R, Chang WC, Aguilar B, Alizadeh D, Wright SL, et al. Chlorotoxin-directed CAR T cells for specific and effective targeting of glioblastoma. Sci Transl Med. 2020;12(533):eaaw2672. 10.1126/scitranslmed.aaw2672. 10.1126/scitranslmed.aaw2672 PMC7500824 32132216

[34] Miao L, Zhang J, Huang B, Zhang Z, Wang S, Tang F (2022). Special chimeric antigen receptor (CAR) modifications of T cells: a review. Front Oncol.

[35] Schneider D, Xiong Y, Wu D, Nӧlle V, Schmitz S, Haso W (2017). A tandem CD19/CD20 CAR lentiviral vector drives on-target and off-target antigen modulation in leukemia cell lines. J Immunother Cancer.

[36] Grada Z, Hegde M, Byrd T, Shaffer DR, Ghazi A, Brawley VS (2013). TanCAR: a novel bispecific chimeric antigen receptor for cancer immunotherapy. Mol Ther Nucleic Acids.

[37] Wang D, Shao Y, Zhang X, Lu G, Liu B (2020). IL-23 and PSMA-targeted duo-CAR T cells in prostate Cancer eradication in a preclinical model. J Transl Med.

[38] Ruella M, Barrett DM, Kenderian SS, Shestova O, Hofmann TJ, Perazzelli J (2016). Dual CD19 and CD123 targeting prevents antigen-loss relapses after CD19-directed immunotherapies. J Clin Invest.

[39] Kloss CC, Condomines M, Cartellieri M, Bachmann M, Sadelain M (2013). Combinatorial antigen recognition with balanced signaling promotes selective tumor eradication by engineered T cells. Nat Biotechnol.

[40] Wilkie S, van Schalkwyk MC, Hobbs S, Davies DM, van der Stegen SJ, Pereira ACP (2012). Dual targeting of ErbB2 and MUC1 in breast cancer using chimeric antigen receptors engineered to provide complementary signaling. J Clin Immunol.

[41] Xie B, Li Z, Zhou J, Wang W. Current Status and Perspectives of Dual-Targeting Chimeric Antigen Receptor T-Cell Therapy for the Treatment of Hematological Malignancies. Cancers (Basel). 2022;14(13):3230. 10.3390/cancers14133230. 10.3390/cancers14133230 PMC9265066 35805001

[42] Qin H, Ramakrishna S, Nguyen S, Fountaine TJ, Ponduri A, Stetler-Stevenson M (2018). Preclinical development of bivalent chimeric antigen receptors targeting both CD19 and CD22. Mol Ther Oncolytics.

[43] Zhang Y, Li J, Lou X, Chen X, Yu Z, Kang L (2021). A prospective investigation of bispecific CD19/22 CAR T cell therapy in patients with relapsed or refractory B cell non-Hodgkin lymphoma. Front Oncol.

[44] Spiegel JY, Patel S, Muffly L, Hossain NM, Oak J, Baird JH (2021). CAR T cells with dual targeting of CD19 and CD22 in adult patients with recurrent or refractory B cell malignancies: a phase 1 trial. Nat Med.

[45] Morsut L, Roybal KT, Xiong X, Gordley RM, Coyle SM, Thomson M (2016). Engineering customized cell sensing and response behaviors using synthetic notch receptors. Cell.

[46] Roybal Kole T, Rupp Levi J, Morsut L, Walker Whitney J, McNally Krista A, Park Jason S (2016). Precision tumor recognition by T cells with combinatorial antigen-sensing circuits. Cell.

[47] Fedorov VD, Themeli M, Sadelain M (2013). PD-1- and CTLA-4-based inhibitory chimeric antigen receptors (iCARs) divert off-target immunotherapy responses. Sci Transl Med.

[48] Yu S, Yi M, Qin S, Wu K (2019). Next generation chimeric antigen receptor T cells: safety strategies to overcome toxicity. Mol Cancer.

[49] Calderon H, Mamonkin M, Guedan S (2020). Analysis of CAR-mediated tonic signaling. Methods Mol Biol.

[50] Long AH, Haso WM, Shern JF, Wanhainen KM, Murgai M, Ingaramo M (2015). 4-1BB costimulation ameliorates T cell exhaustion induced by tonic signaling of chimeric antigen receptors. Nat Med.

[51] Landoni E, Fucá G, Wang J, Chirasani VR, Yao Z, Dukhovlinova E (2021). Modifications to the framework regions eliminate chimeric antigen receptor tonic signaling. Cancer Immunol Res.

[52] Asaadi Y, Jouneghani FF, Janani S, Rahbarizadeh F (2021). A comprehensive comparison between camelid nanobodies and single chain variable fragments. Biomark Res.

[53] Rahbarizadeh F, Rasaee MJ, Forouzandeh Moghadam M, Allameh AA, Sadroddiny E (2004). Production of novel recombinant single-domain antibodies against tandem repeat region of MUC1 mucin. Hybrid Hybridomics.

[54] Arbabi-Ghahroudi M (2017). Camelid single-domain antibodies: historical perspective and future outlook. Front Immunol.

[55] Bakhtiari SH, Rahbarizadeh F, Hasannia S, Ahmadvand D, Iri-Sofla FJ, Rasaee MJ (2009). Anti-MUC1 nanobody can redirect T-body cytotoxic effector function. Hybridoma (Larchmt).

[56] Jamnani FR, Rahbarizadeh F, Shokrgozar MA, Mahboudi F, Ahmadvand D, Sharifzadeh Z (2014). T cells expressing VHH-directed oligoclonal chimeric HER2 antigen receptors: towards tumor-directed oligoclonal T cell therapy. Biochim Biophys Acta.

[57] Hassani M, Hajari Taheri F, Sharifzadeh Z, Arashkia A, Hadjati J, van Weerden WM (2019). Construction of a chimeric antigen receptor bearing a nanobody against prostate a specific membrane antigen in prostate cancer. J Cell Biochem.

[58] Ref. 58: Bao C, Gao Q, Li LL, Han L, Zhang B, Ding Y, et al. The Application of Nanobody in CAR-T Therapy. Biomolecules. 2021;11(2):238. 10.3390/biom11020238. 10.3390/biom11020238 PMC7914546 33567640

[59] Zhao WH, Liu J, Wang BY, Chen YX, Cao XM, Yang Y (2018). A phase 1, open-label study of LCAR-B38M, a chimeric antigen receptor T cell therapy directed against B cell maturation antigen, in patients with relapsed or refractory multiple myeloma. J Hematol Oncol.

[60] Kahlon KS, Brown C, Cooper LJ, Raubitschek A, Forman SJ, Jensen MC (2004). Specific recognition and killing of glioblastoma multiforme by interleukin 13-zetakine redirected cytolytic T cells. Cancer Res.

[61] Moeller R, Scherer J, Kassim S (2021). 871 Construction and evaluation of interleukin 3 (IL3)-zetakine redirected cytolytic T Cells for the treatment of CD123 expressing acute myeloid leukemia. Journal for ImmunoTherapy of Cancer.

[62] Brown CE, Badie B, Barish ME, Weng L, Ostberg JR, Chang WC (2015). Bioactivity and safety of IL13Rα2-redirected chimeric antigen receptor CD8+ T cells in patients with recurrent glioblastoma. Clin Cancer Res.

[63] Wang Y, Xu Y, Li S, Liu J, Xing Y, Xing H (2018). Targeting FLT3 in acute myeloid leukemia using ligand-based chimeric antigen receptor-engineered T cells. J Hematol Oncol.

[64] Maiorova V, Mollaev MD, Vikhreva P, Kulakovskaya E, Pershin D, Chudakov DM, et al. Natural Flt3Lg-Based Chimeric Antigen Receptor (Flt3-CAR) T Cells Successfully Target Flt3 on AML Cell Lines. Vaccines (Basel). 2021;9(11):1238. 10.3390/vaccines9111238. 10.3390/vaccines9111238 PMC8621097 34835169

[65] Baumeister SH, Murad J, Werner L, Daley H, Trebeden-Negre H, Gicobi JK (2019). Phase I trial of autologous CAR T cells targeting NKG2D ligands in patients with AML/MDS and multiple myeloma. Cancer Immunol Res.

[66] Lee L, Draper B, Chaplin N, Philip B, Chin M, Galas-Filipowicz D (2018). An APRIL-based chimeric antigen receptor for dual targeting of BCMA and TACI in multiple myeloma. Blood.

[67] Nakazawa Y, Matsuda K, Kurata T, Sueki A, Tanaka M, Sakashita K (2016). Anti-proliferative effects of T cells expressing a ligand-based chimeric antigen receptor against CD116 on CD34(+) cells of juvenile myelomonocytic leukemia. J Hematol Oncol.

[68] Yan BX, Sun YQ (1997). Glycine residues provide flexibility for enzyme active sites. J Biol Chem.

[69] Chen X, Zaro JL, Shen WC (2013). Fusion protein linkers: property, design and functionality. Adv Drug Deliv Rev.

[70] van Rosmalen M, Krom M, Merkx M (2017). Tuning the flexibility of Glycine-serine linkers to allow rational Design of Multidomain Proteins. Biochemistry.

[71] Hudson PJ, Kortt AA (1999). High avidity scFv multimers; diabodies and triabodies. J Immunol Methods.

[72] Whitlow M, Filpula D, Rollence ML, Feng SL, Wood JF (1994). Multivalent Fvs: characterization of single-chain Fv oligomers and preparation of a bispecific Fv. Protein Eng.

[73] Dolezal O, Pearce LA, Lawrence LJ, McCoy AJ, Hudson PJ, Kortt AA (2000). ScFv multimers of the anti-neuraminidase antibody NC10: shortening of the linker in single-chain Fv fragment assembled in V(L) to V(H) orientation drives the formation of dimers, trimers, tetramers and higher molecular mass multimers. Protein Eng.

[74] Schirrmann T, Menzel C, Hust M, Prilop J, Jostock T, Dübel S (2010). Oligomeric forms of single chain immunoglobulin (scIgG). MAbs.

[75] Shah NN, Highfill SL, Shalabi H, Yates B, Jin J, Wolters PL (2020). CD4/CD8 T-cell selection affects chimeric antigen receptor (CAR) T-cell potency and toxicity: updated results from a phase I anti-CD22 CAR T-cell trial. J Clin Oncol.

[76] Singh N, Frey NV, Engels B, Barrett DM, Shestova O, Ravikumar P (2021). Antigen-independent activation enhances the efficacy of 4-1BB-costimulated CD22 CAR T cells. Nat Med.

[77] Moritz D, Groner B (1995). A spacer region between the single chain antibody- and the CD3 zeta-chain domain of chimeric T cell receptor components is required for efficient ligand binding and signaling activity. Gene Ther.

[78] Qin L, Lai Y, Zhao R, Wei X, Weng J, Lai P (2017). Incorporation of a hinge domain improves the expansion of chimeric antigen receptor T cells. J Hematol Oncol.

[79] Alabanza L, Pegues M, Geldres C, Shi V, Wiltzius JJW, Sievers SA (2017). Function of novel anti-CD19 chimeric antigen receptors with human variable regions is affected by hinge and transmembrane domains. Mol Ther.

[80] Muller YD, Nguyen DP, Ferreira LMR, Ho P, Raffin C, Valencia RVB (2021). The CD28-transmembrane domain mediates chimeric antigen receptor Heterodimerization with CD28. Front Immunol.

[81] Ying Z, Huang XF, Xiang X, Liu Y, Kang X, Song Y (2019). A safe and potent anti-CD19 CAR T cell therapy. Nat Med.

[82] Jensen MC, Popplewell L, Cooper LJ, DiGiusto D, Kalos M, Ostberg JR (2010). Antitransgene rejection responses contribute to attenuated persistence of adoptively transferred CD20/CD19-specific chimeric antigen receptor redirected T cells in humans. Biol Blood Marrow Transplant.

[83] Till BG, Jensen MC, Wang J, Qian X, Gopal AK, Maloney DG (2012). CD20-specific adoptive immunotherapy for lymphoma using a chimeric antigen receptor with both CD28 and 4-1BB domains: pilot clinical trial results. Blood.

[84] Savoldo B, Ramos CA, Liu E, Mims MP, Keating MJ, Carrum G (2011). CD28 costimulation improves expansion and persistence of chimeric antigen receptor-modified T cells in lymphoma patients. J Clin Invest.

[85] Brown CE, Alizadeh D, Starr R, Weng L, Wagner JR, Naranjo A (2016). Regression of glioblastoma after chimeric antigen receptor T-cell therapy. N Engl J Med.

[86] Guest RD, Hawkins RE, Kirillova N, Cheadle EJ, Arnold J, O'Neill A (2005). The role of extracellular spacer regions in the optimal design of chimeric immune receptors: evaluation of four different scFvs and antigens. J Immunother.

[87] Hudecek M, Lupo-Stanghellini MT, Kosasih PL, Sommermeyer D, Jensen MC, Rader C (2013). Receptor affinity and extracellular domain modifications affect tumor recognition by ROR1-specific chimeric antigen receptor T cells. Clin Cancer Res.

[88] Gargett T, Yu W, Dotti G, Yvon ES, Christo SN, Hayball JD (2016). GD2-specific CAR T cells undergo potent activation and deletion following antigen encounter but can be protected from activation-induced cell death by PD-1 blockade. Mol Ther.

[89] Hombach A, Hombach AA, Abken H (2010). Adoptive immunotherapy with genetically engineered T cells: modification of the IgG1 Fc ‘spacer’ domain in the extracellular moiety of chimeric antigen receptors avoids ‘off-target’ activation and unintended initiation of an innate immune response. Gene Ther.

[90] Strohl WR (2009). Optimization of fc-mediated effector functions of monoclonal antibodies. Curr Opin Biotechnol.

[91] Jonnalagadda M, Mardiros A, Urak R, Wang X, Hoffman LJ, Bernanke A (2015). Chimeric antigen receptors with mutated IgG4 fc spacer avoid fc receptor binding and improve T cell persistence and antitumor efficacy. Mol Ther.

[92] Watanabe N, Bajgain P, Sukumaran S, Ansari S, Heslop HE, Rooney CM (2016). Fine-tuning the CAR spacer improves T-cell potency. Oncoimmunology.

[93] Hudecek M, Sommermeyer D, Kosasih PL, Silva-Benedict A, Liu L, Rader C (2015). The nonsignaling extracellular spacer domain of chimeric antigen receptors is decisive for in vivo antitumor activity. Cancer Immunol Res.

[94] Schäfer D, Henze J, Pfeifer R, Schleicher A, Brauner J, Mockel-Tenbrinck N (2020). A novel Siglec-4 derived spacer improves the functionality of CAR T cells against membrane-proximal epitopes. Front Immunol.

[95] Turtle CJ, Hanafi LA, Berger C, Gooley TA, Cherian S, Hudecek M (2016). CD19 CAR-T cells of defined CD4+:CD8+ composition in adult B cell ALL patients. J Clin Invest.

[96] Bister A, Ibach T, Haist C, Smorra D, Roellecke K, Wagenmann M (2021). A novel CD34-derived hinge for rapid and efficient detection and enrichment of CAR T cells. Mol Ther Oncolytics.

[97] Aldoss I, Bargou RC, Nagorsen D, Friberg GR, Baeuerle PA, Forman SJ (2017). Redirecting T cells to eradicate B-cell acute lymphoblastic leukemia: bispecific T-cell engagers and chimeric antigen receptors. Leukemia.

[98] Rafiq S, Hackett CS, Brentjens RJ (2020). Engineering strategies to overcome the current roadblocks in CAR T cell therapy. Nat Rev Clin Oncol.

[99] Singh N, Frey NV, Grupp SA, Maude SL (2016). CAR T cell therapy in acute lymphoblastic leukemia and potential for chronic lymphocytic leukemia. Curr Treat Options Oncol.

[100] Zhang A, Sun Y, Du J, Dong Y, Pang H, Ma L, et al. Reducing Hinge Flexibility of CAR-T Cells Prolongs Survival In Vivo With Low Cytokines Release. Front Immunol. 2021;12:724211. 10.3389/fimmu.2021.724211. 10.3389/fimmu.2021.724211 PMC8524077 34675920

[101] Casucci M, Falcone L, Camisa B, Norelli M, Porcellini S, Stornaiuolo A (2018). Extracellular NGFR spacers allow efficient tracking and enrichment of fully functional CAR-T cells co-expressing a suicide gene. Front Immunol.

[102] Iri-Sofla FJ, Rahbarizadeh F, Ahmadvand D, Rasaee MJ (2011). Nanobody-based chimeric receptor gene integration in Jurkat cells mediated by φC31 integrase. Exp Cell Res.

[103] Sharifzadeh Z, Rahbarizadeh F, Shokrgozar MA, Ahmadvand D, Mahboudi F, Jamnani FR (2013). Genetically engineered T cells bearing chimeric nanoconstructed receptors harboring TAG-72-specific camelid single domain antibodies as targeting agents. Cancer Lett.

[104] Fujiwara K, Tsunei A, Kusabuka H, Ogaki E, Tachibana M, Okada N. Hinge and Transmembrane Domains of Chimeric Antigen Receptor Regulate Receptor Expression and Signaling Threshold. Cells. 2020;9(5):1182. 10.3390/cells9051182. 10.3390/cells9051182 PMC7291079 32397414

[105] Bridgeman JS, Hawkins RE, Bagley S, Blaylock M, Holland M, Gilham DE (2010). The optimal antigen response of chimeric antigen receptors harboring the CD3zeta transmembrane domain is dependent upon incorporation of the receptor into the endogenous TCR/CD3 complex. J Immunol.

[106] Zhang T, Wu MR, Sentman CL (2012). An NKp30-based chimeric antigen receptor promotes T cell effector functions and antitumor efficacy in vivo. J Immunol.

[107] Annenkov AE, Moyes SP, Eshhar Z, Mageed RA, Chernajovsky Y (1998). Loss of original antigenic specificity in T cell hybridomas transduced with a chimeric receptor containing single-chain Fv of an anti-collagen antibody and fc epsilonRI-signaling gamma subunit. J Immunol.

[108] Bridgeman JS, Ladell K, Sheard VE, Miners K, Hawkins RE, Price DA (2014). CD3ζ-based chimeric antigen receptors mediate T cell activation via cis- and trans-signalling mechanisms: implications for optimization of receptor structure for adoptive cell therapy. Clin Exp Immunol.

[109] Ferreira LMR, Muller YD (2021). CAR T-cell therapy: is CD28-CAR Heterodimerization its Achilles’ heel?. Front Immunol.

[110] Majzner RG, Rietberg SP, Sotillo E, Dong R, Vachharajani VT, Labanieh L (2020). Tuning the antigen density requirement for CAR T-cell activity. Cancer Discov.

[111] Guedan S, Posey AD, Jr., Shaw C, Wing A, Da T, Patel PR, et al. Enhancing CAR T cell persistence through ICOS and 4-1BB costimulation. JCI Insight. 2018;3(1):e96976. 10.1172/jci.insight.96976. 10.1172/jci.insight.96976 PMC5821198 29321369

[112] Wan Z, Shao X, Ji X, Dong L, Wei J, Xiong Z (2020). Transmembrane domain-mediated Lck association underlies bystander and costimulatory ICOS signaling. Cell Mol Immunol.

[113] Schmidts A, Ormhøj M, Choi BD, Taylor AO, Bouffard AA, Scarfò I (2019). Rational design of a trimeric APRIL-based CAR-binding domain enables efficient targeting of multiple myeloma. Blood Adv.

[114] Wang E, Wang LC, Tsai CY, Bhoj V, Gershenson Z, Moon E (2015). Generation of potent T-cell immunotherapy for CANCER using DAP12-based, multichain, Chimeric Immunoreceptors. Cancer Immunol Res.

[115] Chen L, Flies DB (2013). Molecular mechanisms of T cell co-stimulation and co-inhibition. Nat Rev Immunol.

[116] Jenkins MK, Ashwell JD, Schwartz RH (1988). Allogeneic non-T spleen cells restore the responsiveness of normal T cell clones stimulated with antigen and chemically modified antigen-presenting cells. J Immunol.

[117] Schwartz RH, Mueller DL, Jenkins MK, Quill H (1989). T-cell clonal anergy. Cold Spring Harb Symp Quant Biol.

[118] Eshhar Z, Waks T, Gross G, Schindler DG (1993). Specific activation and targeting of cytotoxic lymphocytes through chimeric single chains consisting of antibody-binding domains and the gamma or zeta subunits of the immunoglobulin and T-cell receptors. Proc Natl Acad Sci U S A.

[119] Hwu P, Shafer GE, Treisman J, Schindler DG, Gross G, Cowherd R (1993). Lysis of ovarian cancer cells by human lymphocytes redirected with a chimeric gene composed of an antibody variable region and the fc receptor gamma chain. J Exp Med.

[120] Hwu P, Yang JC, Cowherd R, Treisman J, Shafer GE, Eshhar Z (1995). In vivo antitumor activity of T cells redirected with chimeric antibody/T-cell receptor genes. Cancer Res.

[121] Kershaw MH, Westwood JA, Parker LL, Wang G, Eshhar Z, Mavroukakis SA (2006). A phase I study on adoptive immunotherapy using gene-modified T cells for ovarian cancer. Clin Cancer Res.

[122] Lim WA, June CH (2017). The principles of engineering immune cells to treat Cancer. Cell.

[123] Watts TH, DeBenedette MA (1999). T cell co-stimulatory molecules other than CD28. Curr Opin Immunol.

[124] Redmond WL, Ruby CE, Weinberg AD (2009). The role of OX40-mediated co-stimulation in T-cell activation and survival. Crit Rev Immunol.

[125] Gmünder H, Lesslauer W (1984). A 45-kDa human T-cell membrane glycoprotein functions in the regulation of cell proliferative responses. Eur J Biochem.

[126] Lesslauer W, Koning F, Ottenhoff T, Giphart M, Goulmy E, van Rood JJ (1986). T90/44 (9.3 antigen). A cell surface molecule with a function in human T cell activation. Eur J Immunol.

[127] Riha P, Rudd CE (2010). CD28 co-signaling in the adaptive immune response. Self Nonself.

[128] van der Merwe PA, Bodian DL, Daenke S, Linsley P, Davis SJ (1997). CD80 (B7-1) binds both CD28 and CTLA-4 with a low affinity and very fast kinetics. J Exp Med.

[129] Harding FA, McArthur JG, Gross JA, Raulet DH, Allison JP (1992). CD28-mediated signalling co-stimulates murine T cells and prevents induction of anergy in T-cell clones. Nature.

[130] Lindstein T, June CH, Ledbetter JA, Stella G, Thompson CB (1989). Regulation of lymphokine messenger RNA stability by a surface-mediated T cell activation pathway. Science.

[131] Fraser JD, Irving BA, Crabtree GR, Weiss A (1991). Regulation of interleukin-2 gene enhancer activity by the T cell accessory molecule CD28. Science.

[132] Boise LH, Minn AJ, Noel PJ, June CH, Accavitti MA, Lindsten T (1995). CD28 costimulation can promote T cell survival by enhancing the expression of Bcl-XL. Immunity.

[133] Noel PJ, Boise LH, Green JM, Thompson CB (1996). CD28 costimulation prevents cell death during primary T cell activation. J Immunol.

[134] Radvanyi LG, Shi Y, Vaziri H, Sharma A, Dhala R, Mills GB (1996). CD28 costimulation inhibits TCR-induced apoptosis during a primary T cell response. J Immunol.

[135] Frauwirth KA, Riley JL, Harris MH, Parry RV, Rathmell JC, Plas DR (2002). The CD28 signaling pathway regulates glucose metabolism. Immunity.

[136] Granelli-Piperno A, Nolan P (1991). Nuclear transcription factors that bind to elements of the IL-2 promoter. Induction requirements in primary human T cells. J Immunol.

[137] Shapiro VS, Truitt KE, Imboden JB, Weiss A (1997). CD28 mediates transcriptional upregulation of the interleukin-2 (IL-2) promoter through a composite element containing the CD28RE and NF-IL-2B AP-1 sites. Mol Cell Biol.

[138] Miller J, Baker C, Cook K, Graf B, Sanchez-Lockhart M, Sharp K (2009). Two pathways of costimulation through CD28. Immunol Res.

[139] Viola A, Schroeder S, Sakakibara Y, Lanzavecchia A (1999). T lymphocyte costimulation mediated by reorganization of membrane microdomains. Science.

[140] Boomer JS, Green JM (2010). An enigmatic tail of CD28 signaling. Cold Spring Harb Perspect Biol.

[141] Zumerle S, Molon B, Viola A. Membrane Rafts in T Cell Activation: A Spotlight on CD28 Costimulation. Front Immunol. 2017;8:1467. 10.3389/fimmu.2017.01467. 10.3389/fimmu.2017.01467 PMC5675840 29163534

[142] Yoshinaga SK, Whoriskey JS, Khare SD, Sarmiento U, Guo J, Horan T (1999). T-cell co-stimulation through B7RP-1 and ICOS. Nature.

[143] Riley JL, Mao M, Kobayashi S, Biery M, Burchard J, Cavet G (2002). Modulation of TCR-induced transcriptional profiles by ligation of CD28, ICOS, and CTLA-4 receptors. Proc Natl Acad Sci U S A.

[144] Coyle AJ, Lehar S, Lloyd C, Tian J, Delaney T, Manning S (2000). The CD28-related molecule ICOS is required for effective T cell-dependent immune responses. Immunity.

[145] Dong C, Juedes AE, Temann UA, Shresta S, Allison JP, Ruddle NH (2001). ICOS co-stimulatory receptor is essential for T-cell activation and function. Nature.

[146] Paulos CM, Carpenito C, Plesa G, Suhoski MM, Varela-Rohena A, Golovina TN (2010). The inducible costimulator (ICOS) is critical for the development of human T(H)17 cells. Sci Transl Med.

[147] Harada Y, Ohgai D, Watanabe R, Okano K, Koiwai O, Tanabe K (2003). A single amino acid alteration in cytoplasmic domain determines IL-2 promoter activation by ligation of CD28 but not inducible costimulator (ICOS). J Exp Med.

[148] Kwon BS, Weissman SM (1989). cDNA sequences of two inducible T-cell genes. Proc Natl Acad Sci U S A.

[149] Schwarz H, Blanco FJ, von Kempis J, Valbracht J, Lotz M (1996). ILA, a member of the human nerve growth factor/tumor necrosis factor receptor family, regulates T-lymphocyte proliferation and survival. Blood.

[150] Vinay DS, Kwon BS (2011). 4-1BB signaling beyond T cells. Cell Mol Immunol.

[151] Cannons JL, Lau P, Ghumman B, DeBenedette MA, Yagita H, Okumura K (2001). 4-1BB ligand induces cell division, sustains survival, and enhances effector function of CD4 and CD8 T cells with similar efficacy. J Immunol.

[152] Hurtado JC, Kim Y-J, Kwon BS (1997). Signals through 4-1BB are costimulatory to previously activated splenic T cells and inhibit activation-induced cell death. J Immunol.

[153] Takahashi C, Mittler RS, Vella AT (1999). Cutting edge: 4-1BB is a bona fide CD8 T cell survival signal. J Immunol.

[154] Shuford WW, Klussman K, Tritchler DD, Loo DT, Chalupny J, Siadak AW (1997). 4-1BB costimulatory signals preferentially induce CD8+ T cell proliferation and lead to the amplification in vivo of cytotoxic T cell responses. J Exp Med.

[155] Myers L, Takahashi C, Mittler RS, Rossi RJ, Vella AT (2003). Effector CD8 T cells possess suppressor function after 4-1BB and toll-like receptor triggering. Proc Natl Acad Sci U S A.

[156] Vinay DS, Cha K, Kwon BS (2006). Dual immunoregulatory pathways of 4-1BB signaling. J Mol Med (Berl).

[157] Croft M, So T, Duan W, Soroosh P (2009). The significance of OX40 and OX40L to T-cell biology and immune disease. Immunol Rev.

[158] Croft M (2003). Co-stimulatory members of the TNFR family: keys to effective T-cell immunity?. Nat Rev Immunol.

[159] Dawicki W, Bertram EM, Sharpe AH, Watts TH (2004). 4-1BB and OX40 act independently to facilitate robust CD8 and CD4 recall responses. J Immunol.

[160] Serghides L, Bukczynski J, Wen T, Wang C, Routy JP, Boulassel MR (2005). Evaluation of OX40 ligand as a costimulator of human antiviral memory CD8 T cell responses: comparison with B7.1 and 4-1BBL. J Immunol.

[161] Hombach AA, Heiders J, Foppe M, Chmielewski M, Abken H (2012). OX40 costimulation by a chimeric antigen receptor abrogates CD28 and IL-2 induced IL-10 secretion by redirected CD4+ T cells. OncoImmunology.

[162] Zhang H, Li F, Cao J, Wang X, Cheng H, Qi K, et al. A chimeric antigen receptor with antigen-independent OX40 signaling mediates potent antitumor activity. Sci Transl Med. 2021;13(578):eaba7308. 10.1126/scitranslmed.aba7308. 10.1126/scitranslmed.aba7308 33504651

[163] Croft M (2009). The role of TNF superfamily members in T-cell function and diseases. Nat Rev Immunol.

[164] Bullock TN (2017). Stimulating CD27 to quantitatively and qualitatively shape adaptive immunity to cancer. Curr Opin Immunol.

[165] He Y, Vlaming M, van Meerten T, Bremer E. The Implementation of TNFRSF Co-Stimulatory Domains in CAR-T Cells for Optimal Functional Activity. Cancers (Basel). 2022;14(2):299. 10.3390/cancers14020299. 10.3390/cancers14020299 PMC8773791 35053463

[166] Cappell KM, Kochenderfer JN (2021). A comparison of chimeric antigen receptors containing CD28 versus 4-1BB costimulatory domains. Nat Rev Clin Oncol.

[167] Schuster SJ, Bishop MR, Tam CS, Waller EK, Borchmann P, McGuirk JP (2019). Tisagenlecleucel in adult relapsed or refractory diffuse large B-cell lymphoma. N Engl J Med.

[168] Abramson JS, Palomba ML, Gordon LI, Lunning MA, Wang M, Arnason J (2020). Lisocabtagene maraleucel for patients with relapsed or refractory large B-cell lymphomas (TRANSCEND NHL 001): a multicentre seamless design study. Lancet.

[169] Milone MC, Fish JD, Carpenito C, Carroll RG, Binder GK, Teachey D (2009). Chimeric receptors containing CD137 signal transduction domains mediate enhanced survival of T cells and increased antileukemic efficacy in vivo. Mol Ther.

[170] Priceman SJ, Gerdts EA, Tilakawardane D, Kennewick KT, Murad JP, Park AK (2018). Co-stimulatory signaling determines tumor antigen sensitivity and persistence of CAR T cells targeting PSCA+ metastatic prostate cancer. Oncoimmunology.

[171] Amatya C, Pegues MA, Lam N, Vanasse D, Geldres C, Choi S (2021). Development of CAR T cells expressing a suicide gene plus a chimeric antigen receptor targeting signaling lymphocytic-activation molecule F7. Mol Ther.

[172] Powell DJ, Dudley ME, Robbins PF, Rosenberg SA (2005). Transition of late-stage effector T cells to CD27+ CD28+ tumor-reactive effector memory T cells in humans after adoptive cell transfer therapy. Blood.

[173] Song D-G, Powell DJ (2012). Pro-survival signaling via CD27 costimulation drives effective CAR T-cell therapy. Oncoimmunology.

[174] Locke FL, Neelapu SS, Bartlett NL, Siddiqi T, Chavez JC, Hosing CM (2017). Phase 1 results of ZUMA-1: a multicenter study of KTE-C19 anti-CD19 CAR T cell therapy in refractory aggressive lymphoma. Mol Ther.

[175] Porter DL, Hwang WT, Frey NV, Lacey SF, Shaw PA, Loren AW (2015). Chimeric antigen receptor T cells persist and induce sustained remissions in relapsed refractory chronic lymphocytic leukemia. Sci Transl Med.

[176] Song DG, Ye Q, Poussin M, Harms GM, Figini M, Powell DJ (2012). CD27 costimulation augments the survival and antitumor activity of redirected human T cells in vivo. Blood.

[177] Wutti-In Y, Sujjitjoon J, Sawasdee N, Panya A, Kongkla K, Yuti P (2021). Development of a novel anti-CD19 CAR containing a fully human scFv and three costimulatory domains. Front Oncol.

[178] Kawalekar OU, O'Connor RS, Fraietta JA, Guo L, McGettigan SE, Posey AD (2016). Distinct signaling of Coreceptors regulates specific metabolism pathways and impacts memory development in CAR T cells. Immunity.

[179] Zhao Z, Condomines M, van der Stegen SJC, Perna F, Kloss CC, Gunset G (2015). Structural Design of Engineered Costimulation Determines Tumor Rejection Kinetics and Persistence of CAR T cells. Cancer Cell.

[180] Guedan S, Madar A, Casado-Medrano V, Shaw C, Wing A, Liu F (2020). Single residue in CD28-costimulated CAR-T cells limits long-term persistence and antitumor durability. J Clin Invest.

[181] Wang M, Munoz J, Goy A, Locke FL, Jacobson CA, Hill BT (2020). KTE-X19 CAR T-cell therapy in relapsed or refractory mantle-cell lymphoma. N Engl J Med.

[182] Brudno JN, Lam N, Vanasse D, Shen YW, Rose JJ, Rossi J (2020). Safety and feasibility of anti-CD19 CAR T cells with fully human binding domains in patients with B-cell lymphoma. Nat Med.

[183] Munroe ME, Bishop GA (2007). A costimulatory function for T cell CD40. J Immunol.

[184] Nunoya JI, Masuda M, Ye C, Su L (2019). Chimeric antigen receptor T cell bearing herpes virus entry mediator co-stimulatory signal domain exhibits high functional potency. Mol Ther Oncolytics.

[185] Golubovskaya VM, Berahovich R, Xu Q, Zhou H, Xu S, Guan J (2018). GITR domain inside CAR co-stimulates activity of CAR-T cells against cancer. Front Biosci (Landmark Ed).

[186] Collinson-Pautz MR, Chang WC, Lu A, Khalil M, Crisostomo JW, Lin PY (2019). Constitutively active MyD88/CD40 costimulation enhances expansion and efficacy of chimeric antigen receptor T cells targeting hematological malignancies. Leukemia.

[187] Lai Y, Weng J, Wei X, Qin L, Lai P, Zhao R (2018). Toll-like receptor 2 costimulation potentiates the antitumor efficacy of CAR T cells. Leukemia.

[188] Liang X, Huang Y, Li D, Yang X, Jiang L, Zhou W, et al. Distinct functions of CAR-T cells possessing a dectin-1 intracellular signaling domain. Gene Ther. 2021. 10.1038/s41434-021-00257-7. 10.1038/s41434-021-00257-7 33953316

[189] Sadelain M, Brentjens R, Rivière I (2009). The promise and potential pitfalls of chimeric antigen receptors. Curr Opin Immunol.

[190] Hombach A, Muche JM, Gerken M, Gellrich S, Heuser C, Pohl C (2001). T cells engrafted with a recombinant anti-CD30 receptor target autologous CD30(+) cutaneous lymphoma cells. Gene Ther.

[191] Feucht J, Sun J, Eyquem J, Ho YJ, Zhao Z, Leibold J (2019). Calibration of CAR activation potential directs alternative T cell fates and therapeutic potency. Nat Med.

[192] Fisher J, Abramowski P, Wisidagamage Don ND, Flutter B, Capsomidis A, Cheung GW (2017). Avoidance of on-target off-tumor activation using a co-stimulation-only chimeric antigen receptor. Mol Ther.

[193] Zufferey R, Dull T, Mandel RJ, Bukovsky A, Quiroz D, Naldini L (1998). Self-inactivating lentivirus vector for safe and efficient in vivo gene delivery. J Virol.

[194] Sertkaya H, Ficarelli M, Sweeney NP, Parker H, Vink CA, Swanson CM (2021). HIV-1 sequences in lentiviral vector genomes can be substantially reduced without compromising transduction efficiency. Sci Rep.

[195] Powell SK, Rivera-Soto R, Gray SJ (2015). Viral expression cassette elements to enhance transgene target specificity and expression in gene therapy. Discov Med.

[196] Rad SMAH, Poudel A, Tan GMY, McLellan AD (2020). Promoter choice: who should drive the CAR in T cells?. Plos One.

[197] Isomura H, Stinski MF (2003). The human cytomegalovirus major immediate-early enhancer determines the efficiency of immediate-early gene transcription and viral replication in permissive cells at low multiplicity of infection. J Virol.

[198] Fang Y, Zhang Y, Guo C, Chen C, Gao H, Zhou X (2020). Safety and efficacy of an immune cell-specific chimeric promoter in regulating anti-PD-1 antibody expression in CAR T cells. Mol Ther Methods Clin Dev.

[199] Frigault MJ, Lee J, Basil MC, Carpenito C, Motohashi S, Scholler J (2015). Identification of chimeric antigen receptors that mediate constitutive or inducible proliferation of T cells. Cancer Immunol Res.

[200] Kochenderfer JN, Feldman SA, Zhao Y, Xu H, Black MA, Morgan RA (2009). Construction and preclinical evaluation of an anti-CD19 chimeric antigen receptor. J Immunother.

[201] Hughes MS, Yu YY, Dudley ME, Zheng Z, Robbins PF, Li Y (2005). Transfer of a TCR gene derived from a patient with a marked antitumor response conveys highly active T-cell effector functions. Hum Gene Ther.

[202] Brandt LJB, Barnkob MB, Michaels YS, Heiselberg J, Barington T (2020). Emerging approaches for regulation and control of CAR T cells: a Mini review. Front Immunol.

[203] Eyquem J, Mansilla-Soto J, Giavridis T, van der Stegen SJC, Hamieh M, Cunanan KM (2017). Targeting a CAR to the TRAC locus with CRISPR/Cas9 enhances tumour rejection. Nature.

[204] Klump H, Schiedlmeier B, Vogt B, Ryan M, Ostertag W, Baum C (2001). Retroviral vector-mediated expression of HoxB4 in hematopoietic cells using a novel coexpression strategy. Gene Ther.

[205] Martínez-Salas E (1999). Internal ribosome entry site biology and its use in expression vectors. Curr Opin Biotechnol.

[206] Mizuguchi H, Xu Z, Ishii-Watabe A, Uchida E, Hayakawa T (2000). IRES-dependent second gene expression is significantly lower than cap-dependent first gene expression in a bicistronic vector. Mol Ther.

[207] Emerman M, Temin HM (1986). Quantitative analysis of gene suppression in integrated retrovirus vectors. Mol Cell Biol.

[208] Curtin JA, Dane AP, Swanson A, Alexander IE, Ginn SL (2008). Bidirectional promoter interference between two widely used internal heterologous promoters in a late-generation lentiviral construct. Gene Ther.

[209] Amendola M, Venneri MA, Biffi A, Vigna E, Naldini L (2005). Coordinate dual-gene transgenesis by lentiviral vectors carrying synthetic bidirectional promoters. Nat Biotechnol.

[210] He K, Rad SMAH, Poudel A, McLellan AD. Compact Bidirectional Promoters for Dual-Gene Expression in a Sleeping Beauty Transposon. Int J Mol Sci. 2020;21(23):9256. 10.3390/ijms21239256. 10.3390/ijms21239256 PMC7731152 33291599

[211] Mølhøj M, Degan FD (2004). Leader sequences are not signal peptides. Nat Biotechnol.

[212] von Heijne G (1985). Signal sequences. The limits of variation. J Mol Biol.

[213] Owji H, Nezafat N, Negahdaripour M, Hajiebrahimi A, Ghasemi Y (2018). A comprehensive review of signal peptides: structure, roles, and applications. Eur J Cell Biol.

[214] Walter P, Blobel G (1980). Purification of a membrane-associated protein complex required for protein translocation across the endoplasmic reticulum. Proc Natl Acad Sci.

[215] Kapp K SS, Lemberg MK, et al. Post-Targeting functions of signal peptides. madame curie bioscience database, Austin: Landes Bioscience; 2000-2013, Available from https://www.ncbi.nlm.nih.gov/books/NBK6322/.

[216] Walter P, Gilmore R, Blobel G (1984). Protein translocation across the endoplasmic reticulum. Cell.

[217] Zhang E, Gu J, Xue J, Lin C, Liu C, Li M (2018). Accurate control of dual-receptor-engineered T cell activity through a bifunctional anti-angiogenic peptide. J Hematol Oncol.

[218] Moot R, Raikar SS, Fleischer L, Querrey M, Tylawsky DE, Nakahara H (2016). Genetic engineering of chimeric antigen receptors using lamprey derived variable lymphocyte receptors. Mol Ther Oncolytics.

[219] Cooper LJ, Topp MS, Serrano LM, Gonzalez S, Chang WC, Naranjo A (2003). T-cell clones can be rendered specific for CD19: toward the selective augmentation of the graft-versus-B-lineage leukemia effect. Blood.

[220] Feldmann A, Hoffmann A, Bergmann R, Koristka S, Berndt N, Arndt C (2020). Versatile chimeric antigen receptor platform for controllable and combinatorial T cell therapy. OncoImmunology.

[221] Wang X, Chang WC, Wong CW, Colcher D, Sherman M, Ostberg JR (2011). A transgene-encoded cell surface polypeptide for selection, in vivo tracking, and ablation of engineered cells. Blood.

[222] Güler-Gane G, Kidd S, Sridharan S, Vaughan TJ, Wilkinson TC, Tigue NJ (2016). Overcoming the refractory expression of secreted recombinant proteins in mammalian cells through modification of the signal peptide and adjacent amino acids. Plos One.

[223] Ping Y, Li F, Nan S, Zhang D, Shi X, Shan J (2020). Augmenting the effectiveness of CAR-T cells by enhanced self-delivery of PD-1-neutralizing scFv. Front Cell Dev Biol.

[224] Lee J-H, Culver G, Carpenter S, Dobbs D (2008). Analysis of the EIAV rev-responsive element (RRE) reveals a conserved RNA motif required for high affinity rev binding in both HIV-1 and EIAV. Plos One.

[225] Fernandes J, Jayaraman B, Frankel A (2012). The HIV-1 rev response element: an RNA scaffold that directs the cooperative assembly of a homo-oligomeric ribonucleoprotein complex. RNA Biol.

[226] Zapp ML, Green MR (1989). Sequence-specific RNA binding by the HIV-1 rev protein. Nature.

[227] Sherpa C, Rausch JW, Le Grice SF, Hammarskjold ML, Rekosh D (2015). The HIV-1 rev response element (RRE) adopts alternative conformations that promote different rates of virus replication. Nucleic Acids Res.

[228] DiMattia MA, Watts NR, Cheng N, Huang R, Heymann JB, Grimes JM (2016). The structure of HIV-1 rev filaments suggests a bilateral model for rev-RRE assembly. Structure.

[229] Hoffmann D, Schwarck D, Banning C, Brenner M, Mariyanna L, Krepstakies M (2012). Formation of trans-activation competent HIV-1 rev:RRE complexes requires the recruitment of multiple protein activation domains. Plos One.

[230] Booth DS, Cheng Y, Frankel AD (2014). The export receptor Crm1 forms a dimer to promote nuclear export of HIV RNA. Elife.

[231] Daugherty MD, Booth DS, Jayaraman B, Cheng Y, Frankel AD (2010). HIV rev response element (RRE) directs assembly of the rev homooligomer into discrete asymmetric complexes. Proc Natl Acad Sci U S A.

[232] Wurtzer S, Goubard A, Mammano F, Saragosti S, Lecossier D, Hance AJ (2006). Functional central polypurine tract provides downstream protection of the human immunodeficiency virus type 1 genome from editing by APOBEC3G and APOBEC3B. J Virol.

[233] Charneau P, Clavel F (1991). A single-stranded gap in human immunodeficiency virus unintegrated linear DNA defined by a central copy of the polypurine tract. J Virol.

[234] Charneau P, Alizon M, Clavel F (1992). A second origin of DNA plus-strand synthesis is required for optimal human immunodeficiency virus replication. J Virol.

[235] Arhel N, Munier S, Souque P, Mollier K, Charneau P (2006). Nuclear import defect of human immunodeficiency virus type 1 DNA flap mutants is not dependent on the viral strain or target cell type. J Virol.

[236] Arhel NJ, Souquere-Besse S, Munier S, Souque P, Guadagnini S, Rutherford S (2007). HIV-1 DNA flap formation promotes uncoating of the pre-integration complex at the nuclear pore. EMBO J.

[237] Manganini M, Serafini M, Bambacioni F, Casati C, Erba E, Follenzi A (2002). A human immunodeficiency virus type 1 pol gene-derived sequence (cPPT/CTS) increases the efficiency of transduction of human nondividing monocytes and T lymphocytes by lentiviral vectors. Hum Gene Ther.

[238] Johnson NM, Alvarado AF, Moffatt TN, Edavettal JM, Swaminathan TA, Braun SE (2021). HIV-based lentiviral vectors: origin and sequence differences. Mol Ther Methods Clin Dev.

[239] Van Maele B, De Rijck J, De Clercq E, Debyser Z (2003). Impact of the central polypurine tract on the kinetics of human immunodeficiency virus type 1 vector transduction. J Virol.

[240] Guntaka RV (1993). Transcription termination and polyadenylation in retroviruses. Microbiol Rev.

[241] Furger A, Monks J, Proudfoot NJ (2001). The retroviruses human immunodeficiency virus type 1 and Moloney murine leukemia virus adopt radically different strategies to regulate promoter-proximal polyadenylation. J Virol.

[242] Brown PH, Tiley LS, Cullen BR (1991). Efficient polyadenylation within the human immunodeficiency virus type 1 long terminal repeat requires flanking U3-specific sequences. J Virol.

[243] Gilmartin GM, Fleming ES, Oetjen J, Graveley BR (1995). CPSF recognition of an HIV-1 mRNA 3′-processing enhancer: multiple sequence contacts involved in poly(a) site definition. Genes Dev.

[244] Schambach A, Galla M, Maetzig T, Loew R, Baum C (2007). Improving transcriptional termination of self-inactivating gamma-retroviral and lentiviral vectors. Mol Ther.

[245] Hager S, Frame FM, Collins AT, Burns JE, Maitland NJ (2008). An internal polyadenylation signal substantially increases expression levels of lentivirus-delivered transgenes but has the potential to reduce viral titer in a promoter-dependent manner. Hum Gene Ther.

[246] Choi J-H, Yu N-K, Baek G-C, Bakes J, Seo D, Nam HJ (2014). Optimization of AAV expression cassettes to improve packaging capacity and transgene expression in neurons. Mol Brain.

[247] Donello JE, Loeb JE, Hope TJ (1998). Woodchuck hepatitis virus contains a tripartite posttranscriptional regulatory element. J Virol.

[248] Zufferey R, Donello JE, Trono D, Hope TJ (1999). Woodchuck hepatitis virus posttranscriptional regulatory element enhances expression of transgenes delivered by retroviral vectors. J Virol.

[249] Loeb JE, Cordier WS, Harris ME, Weitzman MD, Hope TJ (1999). Enhanced expression of transgenes from adeno-associated virus vectors with the woodchuck hepatitis virus posttranscriptional regulatory element: implications for gene therapy. Hum Gene Ther.

[250] Xu ZL, Mizuguchi H, Mayumi T, Hayakawa T (2003). Woodchuck hepatitis virus post-transcriptional regulation element enhances transgene expression from adenovirus vectors. Biochim Biophys Acta.

[251] Higashimoto T, Urbinati F, Perumbeti A, Jiang G, Zarzuela A, Chang LJ (2007). The woodchuck hepatitis virus post-transcriptional regulatory element reduces readthrough transcription from retroviral vectors. Gene Ther.

[252] Zaiss AK, Son S, Chang LJ (2002). RNA 3′ readthrough of oncoretrovirus and lentivirus: implications for vector safety and efficacy. J Virol.

[253] Iwakuma T, Cui Y, Chang LJ (1999). Self-inactivating lentiviral vectors with U3 and U5 modifications. Virology.

[254] Yang Q, Lucas A, Son S, Chang L-J (2007). Overlapping enhancer/promoter and transcriptional termination signals in the lentiviral long terminal repeat. Retrovirology.

[255] Lukjanov V, Koutná I, Šimara P (2021). CAR T-cell production using nonviral approaches. J Immunol Res.

[256] Singh H, Moyes JS, Huls MH, Cooper LJ (2015). Manufacture of T cells using the sleeping beauty system to enforce expression of a CD19-specific chimeric antigen receptor. Cancer Gene Ther.

[257] Cary LC, Goebel M, Corsaro BG, Wang HG, Rosen E, Fraser MJ (1989). Transposon mutagenesis of baculoviruses: analysis of Trichoplusia ni transposon IFP2 insertions within the FP-locus of nuclear polyhedrosis viruses. Virology.

[258] Tsukahara T, Iwase N, Kawakami K, Iwasaki M, Yamamoto C, Ohmine K (2015). The Tol2 transposon system mediates the genetic engineering of T-cells with CD19-specific chimeric antigen receptors for B-cell malignancies. Gene Ther.

[259] Kebriaei P, Singh H, Huls MH, Figliola MJ, Bassett R, Olivares S (2016). Phase I trials using sleeping beauty to generate CD19-specific CAR T cells. J Clin Invest.

[260] Kebriaei P, Huls H, Neel SL, Olivares S, Orozco AF, Su S (2017). Shortening the time to manufacture CAR+ T cells with sleeping beauty system supports T-cell engraftment and anti-tumor effects in patients with refractory CD19+ tumors. Blood.

[261] Magnani CF, Gaipa G, Belotti D, Matera G, Tettamanti S, Cabiati B (2019). Donor-derived CD19 CAR cytokine induced killer (CIK) cells engineered with sleeping beauty transposon for relapsed B-cell acute lymphoblastic leukemia (B-ALL). Blood.

[262] Li C, Sun Y, Wang J, Tang L, Jiang H, Guo T (2021). PiggyBac-generated CAR19-T cells plus Lenalidomide cause durable complete remission of triple-hit refractory/relapsed DLBCL: a case report. Front Immunol.

[263] Pomeroy EJ, Lahr WS, Chang JW, Krueger JB, Wick BJ, Slipek NJ, et al.. Non-Viral Engineering of CAR-NK and CAR-T Cells Using the Tc Buster Transposon System™. bioRxiv. 2021. 10.1101/2021.08.02.454772.

[264] Tipanee J, Samara-Kuko E, Gevaert T, Chuah MK, VandenDriessche T. Universal allogeneic CAR T cells engineered with Sleeping Beauty transposons and CRISPR-CAS9 for cancer immunotherapy. Mol Ther. 2022:S1525-0016(22)00366-5. 10.1016/j.ymthe.2022.06.006. 10.1016/j.ymthe.2022.06.006 PMC9552804 35711141

